# Supplementary material for: Leishmania protein KMP-11 modulates cholesterol transport and membrane fluidity to facilitate host cell invasion
Source: EMBO Rep. 2024 Oct 31;25(12):5561–98. doi: 10.1038/s44319-024-00302-7 (PMC11624268; doi:10.1038/s44319-024-00302-7)
Supplement: Supplementary file 1 — Appendix [file 44319_2024_302_MOESM1_ESM.pdf]

Appendix

Table of contents

	Page No.
Appendix Methods	2-5
Appendix Table S1	6
Appendix Table S2	7
Appendix Table S3	8
Appendix Table S4	9
Appendix Table S5	10
Appendix Table S6	11-12
Appendix Table S7	13
Appendix Figure S1	14
Appendix Figure S2	15-16
Appendix Figure S3	17
Appendix Figure S4	18-19
Appendix Figure S5	20
Appendix Figure S6	21-22
Appendix References	23

Appendix Methods.

Evaluation of critical P/L ratio for phase transition of MΦ membrane during LD infection:

To understand the availability of sufficient KMP-11 molecules on L.donovani surface for macrophage membrane interactions and subsequent phase transition we evaluated the approximate number of KMP-11 based on our experimental results. It has been found that at P/L ratio> 0.004, DPPC remains at solution (Fluid phase) phase at physiological temperature (37°C).

Now we want to quantify the number of KMP-11 copy numbers essential for inducing the phase state change of host macrophage membranes at physiological condition.

It is known that lipid head group area[1] = 63 $Å^2$

Our as prepared Vesicles (SUVs) have radius ~ 70 nm.

Then area of a vesicle = $4\pi r^2$ = 61544 nm^2.

Vesicles consist of bilayers. Assuming layer similar (layer approximation), the overall area would be = 61544 X 2= 123088 nm^2.

Then, no. of lipid molecules (fatty acids) in a vesicle = 123088 nm^2/63 $Å^2$ = 195378

Now, the working concentration of DPPC lipid = 1 mM.

So, no. of vesicles in 1 mM = 6.023 X10^{20}/195378 =3.08 X10^{15}

The number of vesicles in our working solution = 3.08 X10^{15}

On the other hand, protein concentration = 4 μM (since, we have taken P/L = 0.004 and in this concentration phase transition occurred at below physiological temperature).

Now, 4 μM of KMP-11 is equivalent to 6.023X10^{23}. 4 X 10^{-6} = 2.4 X 10^{18} number of KMP-11

Therefore, in working (favorable for infection through phase transition) condition,

No. of proteins needed/ lipid vesicles = 2.4 X10^{18}/3.08 X10^{15}= 800

Considering the average size of macrophages = 5 μm which will consist of 1.25 X 10^{7} fatty acids. Now, 195378 fatty acids correspond to 800 number of KMP-11

So, 1.25 X 10^{7}fatty acids correspond to [1.25 X 10^{7} / 195378].800.

~ 5 X 10^{4} copy number of KMP-11.

According to the reports, the expressed copy number of KMP-11/ LD parasite surface is.

~ 2 X 10^{6}.

So, considerable amount of KMP-11 is available for the interaction and inducing infection.

Mathematical exposition of '***Hydrophobic moment and sequence symmetry-oriented phase transition model***':

Protein–lipid interactions are driven by several factors. Among these, hydrophobic interaction is the principal driving force. Although it is generally accepted that globular proteins fold with a hydrophobic core and hydrophilic exterior, the centroid of the spatial distribution of amino acid residue provides the origin of moment expansion. Spatial distribution has been described by David Silverman as a two component spherical model where interacting partners had been considered as spheres[2]. Subsequently, we constructed a '***Hydrophobic moment and sequence symmetry-oriented phase transition model***', in which the nature of a helical stretch was theoretically related to the phase melting temperature of phospholipids.

Assuming the density of hydrophobicity is ρ(R) and the radial distance from the center of the sphere is R, it can be written as:

$\rho(R) = \alpha R^{\eta} - \beta R^{\mu}$, ------------------------- (1)

Where, hydrophobic component will contribute an amount $4\pi\alpha R^{\eta}R^{2}dR$ in a shell of width dR, while the contribution of the polar component is $-4\pi\beta R^{\mu}R^{2}dR$.

Assuming the contribution of polar component is significantly less compared to the hydrophobic component, we can write equation (1) as

$$R = [\rho(R)/\alpha]^{1/\eta} \quad \text{------------------------(2)}$$

Jacob Israelachvili and Richard Pashley have shown that a pair interaction free energy (ΔG) abides by the following relation[3]:

$\Delta G_H = -CRDe^{-\frac{D}{D_0}}$ --------------------(3)

Here, hydrophobic interaction is a two-component system. Therefore, for a two component protein –lipid system, $R = {R_1 R_2}/{(R_1 + R_2)}$; where R_1 (Peptide) and R_2 (Lipid) are the radii of

the interacting partners (**Appendix Figure S6: Ei,ii**). Now equation (3) will be, $\Delta G_H = -C\left({R_1 R_2}/{R_1+R_2}\right) De^{-\frac{D}{D_0}}$

$$= -C\left({1}/{(1/R_1 + 1/R_2)}\right) De^{-\frac{D}{D_0}} \text{----------(4)}$$

Since the lipid component is fixed and the second component (membrane interacting domain of the protein; different peptides) is variable, we can rearrange the equation (4) as

$$\Delta G_H = -CR_1 De^{-\frac{D}{D_0}}$$

$$= -CDe^{-\frac{D}{D_0}}\left(\frac{\rho(R_1)}{\alpha}\right)^{\frac{1}{\eta}}$$

$$= -CDe^{-\frac{D}{D_0}}\left(\frac{H}{\alpha A}\right)^{\frac{1}{\eta}} \text{------------------------- (5)}$$

Where, H is the net hydrophobicity and A is the area of hydrophobic face.

From our experimental and theoretical calculations, it has been established that protein-lipid interaction and phase transition is dependent on the hydrophobic moment of the proteins stretch, therefore, equation (4) can be represented as

$$\Delta G_H = -CDe^{-\frac{D}{D_0}}\left(\frac{\mu_H}{\alpha A}\right)^{\frac{1}{\eta}} \text{-- (6)}$$

Using the mirror sequence constituting residue number (N) and different combinations of polar (P) and hydrophobic (H) residues of KMP-11 amino–terminal stretch (1-19) (**Appendix Table S5)**, we showed the linear correlation between the hydrophobic moment, μ_H and N. The linear equation is expressed (**Appendix Figure S6F**) as:

$$\mu_H = 0.018N + 0.227 \text{----------------------------(7)}$$

Now, combining equation (6) and (7), we get

$\varDelta G_H = -CDe^{-\frac{D}{D_0}} \left(\frac{0.018N+0.227}{\alpha A}\right)^{\frac{1}{\eta}}$------------------------ (8)

Since hydrophobic free energy is the strongest component for the phase transition to occur, we can write, $\varDelta G_H = \Delta G_T$; where $\varDelta G_T$ is the free energy change during gel/fluid phase transition. Now, Thermodynamic equation of free energy would be,

$\varDelta G_T = \varDelta H_T - T_T \varDelta S_T$ -------------------------------- (9) where, T_T stands for the phase transition temperature of phospholipid.

Combining equation (8) and (9), we can write,

$$T_T \varDelta S_T = \varDelta H_T + CDe^{-\frac{D}{D_0}} \left(\frac{0.018N+0.227}{\alpha A}\right)^{\frac{1}{\eta}}$$

Or, $T_T = \varDelta H_T / \varDelta S_T + \frac{CDe^{-\frac{D}{D_0}}\left(\frac{0.018N+0.227}{\alpha A}\right)^{\frac{1}{\eta}}}{\varDelta S_T}$ --------------------------------- (10)

Assuming η = 1, we can write equation (10) as

$T_T = \varDelta H_T / \varDelta S_T + \frac{K(0.018N+0.227)}{\varDelta S_T}$ ------------------------------------ (11)

Where, K= $\frac{CDe^{-\frac{D}{D_0}}}{\alpha A}$

Linear fitting **(Appendix Figure S6G)** of the experimental data of transition temperature (T_m) against N has given us simplified linear correlation e.g

$T_m = 42.02 - 0.466N$--- (12)

The proposed model indicated a strong correlation between the sequence amino acid distribution and the protein mediated phase transition temperature of phospholipid membranes.

Appendix Table S1: Fluorescence lifetime (T_{avg}, nsec) of DiD labeled DPPC SUVs in presence and absence of r-KMP-11 at protein/lipid 0.02 (molar ratio). The data were represented as Mean±SD evaluated from three independent experiments.

**Systems**	**T_1**	**T_2**	**T_{avg}**	**X^2**
**DiD -DPPC**	3.397±0.121	5.695±0.152	4.107±0.115	1.091
**DiD-DPPC-r-KMP-11**	2.813±0.098	4.438±0.132	3.968±0.109	1.049

Appendix Table S2: Binding constants (K_a), cooperative index (n) and Stern-Volmer constants (K_{sv}) of different protein mutants with DPPC model membranes. Binding experiments were performed using DiD labeled DPPC SUVs in presence r-KMP-11.

Systems	K_a (M^{-1})	n	K_{sv} (M^{-1})
r-KMP-11-DPPC	3.504 x10^6	1.450	------
Y5W	--------	-------	4.909
Y5W-DPPC	5.154 x10^7	2.117	2.453
F19W	----------	-----------	4.665
F19W-DPPC	4.351 x10^7	1.822	3.146
F30W	----------	--------	5.142
F30W-DPPC	2.200 x10^7	1.772	4.716
Y48W	-----------	--------	1.799
Y48W-DPPC	8.508 x10^6	1.945	1.455
F51W	---------	---------	4.268
F51W-DPPC	1.540 x10^7	1.682	4.103
F62W	-------	-------	3.177
F62W-DPPC	2.674 x10^5	1.562	4.454
F77W	--------	--------	9.862
F77W-DPPC	3.000 x10^6	1.624	9.567
Y89W	--------	---------	4.454
Y89W-DPPC	6.205 x10^6	2.364	4.152

Appendix Table S3: Chain melting temperature of DPPC membrane using different r-KMP-11 protein (P)/DPPC Lipid (L) molar ratio as monitored by measuring the polarsiation of Laurdan and REES of NBD PC.

P/L ratio	Chain melting temperature using laurdan	Chain melting temperature using NBDPC
0	44.8	44.4
0.0002	47.4	46.2
0.001	48.0	43.9
0.002	40.4	36.5
0.0025	39.5	37.7
0.004	38.7	33.9
0.02	33.5	33.2

Appendix Table S4: Efficiency of energy transfer (E) and donor-acceptor distances (r_0) of different tryptophan residues of eight single tryptophan mutants. Here, tryptophan was the donor and DHE in DPPC SUVs was used as the acceptor molecule. The binding of different mutants with DPPC SUVs was examined to determine the orientation of KMP-11 in DPPC membrane.

Mutants	E	r_0 (Å)
Y5W	0.20	11.4
F19W	0.10	12.8
F30W	0.08	18.2
Y48W	0.06	14.1
F51W	0.02	15.6
F62W	0.01	24.7
F77W	0.06	15.8
Y89W	0.08	13.6

Appendix Table S5: Different combinations of the amino acid residues in the amino-terminal stretch (1-19AA) of KMP-11 based on their polarity and hydrophobicity. Here, N denotes the number of amino acid residues and μ_H is the hydrophobic moments calculated by the Helical wheel model. The membrane embedded residues and the free energy changes were calculated in OPM server.

Serial No.	Peptide sequences	N	μH	Embedded residues	ΔG(Kcal/mole)
1	MATTYEEFSAKLDRLDQEF<br>HHPPHPPHPHPHPPHPPPH	15	0.505	1,5,8,12,15,19	-9.7
2	MATTYEEFSAKLDRLFDQE<br>HHPPHPPHPHPHPPHHPPP	11	0.453	1,2,5,8,12	-7.7
3	MATTYEEFSAKLDLRDQEF<br>HHPPHPPHPHPHPPPPHPH	9	0.394	1,5,8,12,19	-8.4
4	MATTYEEFSAKLDLRDQEF<br>HHPPHPPHPHPHPHPPPPH	7	0.354	1-5,8	-7.3
5	MATTYEEFSAKLLDRDFQE<br>HHPPHPPHPHPHHPPPHPP	5	0.306	1-6,8	-6.8
6	MATTYEEFSAKDLRDLFQE<br>HHPPHPPHPHPPHPPHHPP	3	0.292	1-2,4-5,8	-6.6

Appendix Table S6: Disease causing proteins with their membrane binding domains and respective N values.

DISEASE	PROTEIN	MEMBRANE BINDING STRETCH	N
Whooping cough	Adenyl-cyclase toxin of B. pertusis	**LFGRAPEVIARA [HHHHHHPHHHHH]**	11
Diarrhoeal diseases and deep wound infections	Aerolysin	**RLFSLGQGVCGDK [HHHPHHPHHPHPP]**	9
Gas gangrene	Alpha toxin from C. perfringens	**KDNSWYLAYSIPDTGES [PPPPHHHHHPHHPPHPP]**	7
Anthrax	Anthrolysin O	**KVSIGGTTLYPTATISH [PHPHHHPPHHHPHHHPP]**	11
Food poisoning/gastrointestinal illnesses	Clostridium perfringens enterotoxin	**QSLGDGVKDHYVDISL [PPHHPHHPPPHHPHPH]**	11
Cholera	Colicin A	**EVESWVLSGIASSVAL [PHPPHHHPHHHPPHHH]**	13
Respiratory tract infection	Delta toxin	**IRKEENGNVTIITQNNKQ [HHPPPPHPHPHHPPPPPP]**	5
Gas gangrene,	Epsilon toxin	**TTTHTVGTSIQATAKFTVPFNETGVSLT TSYSFANTN [PPPPPHHPPHPHPHPHPHHHPPPHHPH PPPHPHHPPP]**	7
Cardiotoxic effects	Equinatoxin II	**QKDRGPVATGAVGVLAYLMSD [PPPHHHHHPHHHHHHHHHHPP]**	11
Hemolytic activity	Gama hemolysin	**SRTTYSDLIKRMIWPF [PHPPHPPHHPHHHHHH]**	5
Endophthalmitis	Hemolysin BL	**LSEIEQTNNGDTAL [HPPHPPPPPHPPHH]**	13
Hemolytic activity	Hemolysin lectin	**NVLATQTLENTSSQTQE [PHHHPPPHPPPPPPPPP]**	9
Forms cytotoxic pores in CD59-positive cells, promotes adherence to and invasion of human liver cells	Intermedilysin	**ATGLAWEPWRLIYS [HPHHHHPHHHHHHP]**	9
Forms cytotoxic pores in CD59-positive cells, promotes adherence to and invasion of human liver cells	Lectinolysin	**EVFRSATNIG [PHHHPHPPHH]**	5
Kill leucocytes	Leucocidin F	**RWNGFYWAGANY [HHPHHHHHHHPH]**	11
Listeriosis	Listeriolysin O	**ECTGLAWEWWRTVID [PPPHHHHPHHHPHHP]**	7
	Perfringolysin O	**YDVPLTNNINVSIWGTTLYPGSST [HPHHHPPPHPHPHHHPPHHHHPPP]**	5
	Sticholysin II	**SVPFDYNWYSNWWDVK [PHHHPHPHHPPHHPHP]**	7
Disrupts cytoplasmic integrity of erythrocytes, leukocytes, macrophages, platelets, epithelial cells	Streptolysin O	**TGLAWEWWRKV [PHHHHPHHHPH]**	7
Cytotoxic to endothelial cells, epithelial cells, macrophages, and neutrophils	Suilysin	**CTGLAWEWWRTVY [PPHHHHPHHHPHH]**	7
Cholera	Vibrio cholerae cytolysin	**AYKHYYVVGAHQSYH [HHPPHHHHHHPPPHP]**	5
Cholera	Vibrio vulnificus hemolysin	**MAHVTLQSLSNN [HHPHPHPPHPPP]**	5

Severe Acute Respiratory Syndrome	Coronavirus Protein 6	**MFHLVDFQVTIAEILIIIIMRTFRIAIWNLDVIISSIVR [HHPHHPHPHPHPPHHHHHHHPPHPHHHHPHP]**	17
Hepatitis C	HCV NS5A (Hepatitis C virus)	**DTSWLRDVWDWVCTVLSDFRVWLQAKLL [PPPHHPPHHPHHPPHHPPHPHHHPPPHH]**	17
Diptheria	Diptheria toxin	**SLTGTNPVFAGANYAAWAVN [PHPPPPHHHHPHPHHHHHHP]**	11
E. Coli	Cytolysin A	**KTVEVVKNAIETADGLDLYNKYLD [PPHPHHPPHHPPHPPHPHHPPHHP]**	7

Appendix Table S7: Primers of the single tryptophan mutants of KMP-11.

**Tryptophan Mutations**	**Primers (5' → 3')**
Y5W	Fwd: CCATGGCCACCACGTGGGAGGAGTTTTCGGCG<br>Rev: GCCGAAAACTCCTCCCACGTGGTGGCCATGG
Y48W	Fwd: CGAGATGAAGGAGCACTGGCAGAAGTTCGAGCGCAT<br>Rev: ATGCGCTCGAACTTCTCCCAGTGCTCCTTCATCTCG
F62W	Fwd: GCTCGTGCATCTTCTTGTTCCACTTCTCTGTGTGTTCCTT<br>Rev: AAGGAACACACAGAGAAGTGGAACAAGAAGATGCACGAGC
Y89W	Fwd: AGCAGAAGGCTGCGCAGTGGCCGTCCAAGC<br>Rev: GCTTGGACGGCCACTGCGCAGCCTTCTGCT
F19W	Fwd: CCGCCTGGATGAGGAGTGGAACCGGAAGATGCAGG<br>Rev: CCTGCATCTTCCGGTTCCACTCCTCATCCAGGCGG
F30W	Fwd: GAGCAGAACGCCAAGTGGTTTGCGGACAAGCCGG<br>Rev: CCGGCTTGTCCGCAAACCACTTGGCGTTCTGCTC
F51W	Fwd: GGAGCACTACGAGAAGTGGGAGCGCATGATCAAGGA<br>Rev: TCCTTGATCATGCGCTCCCACTTCTCGTAGTGCTCC
F77W	Fwd: CGGAGCACTTCAAGCAGAAGTGGGCCGAGCTGCT<br>Rev: AGCAGCTCGGCCCACTTCTGCTTGAAGTGCTCCG

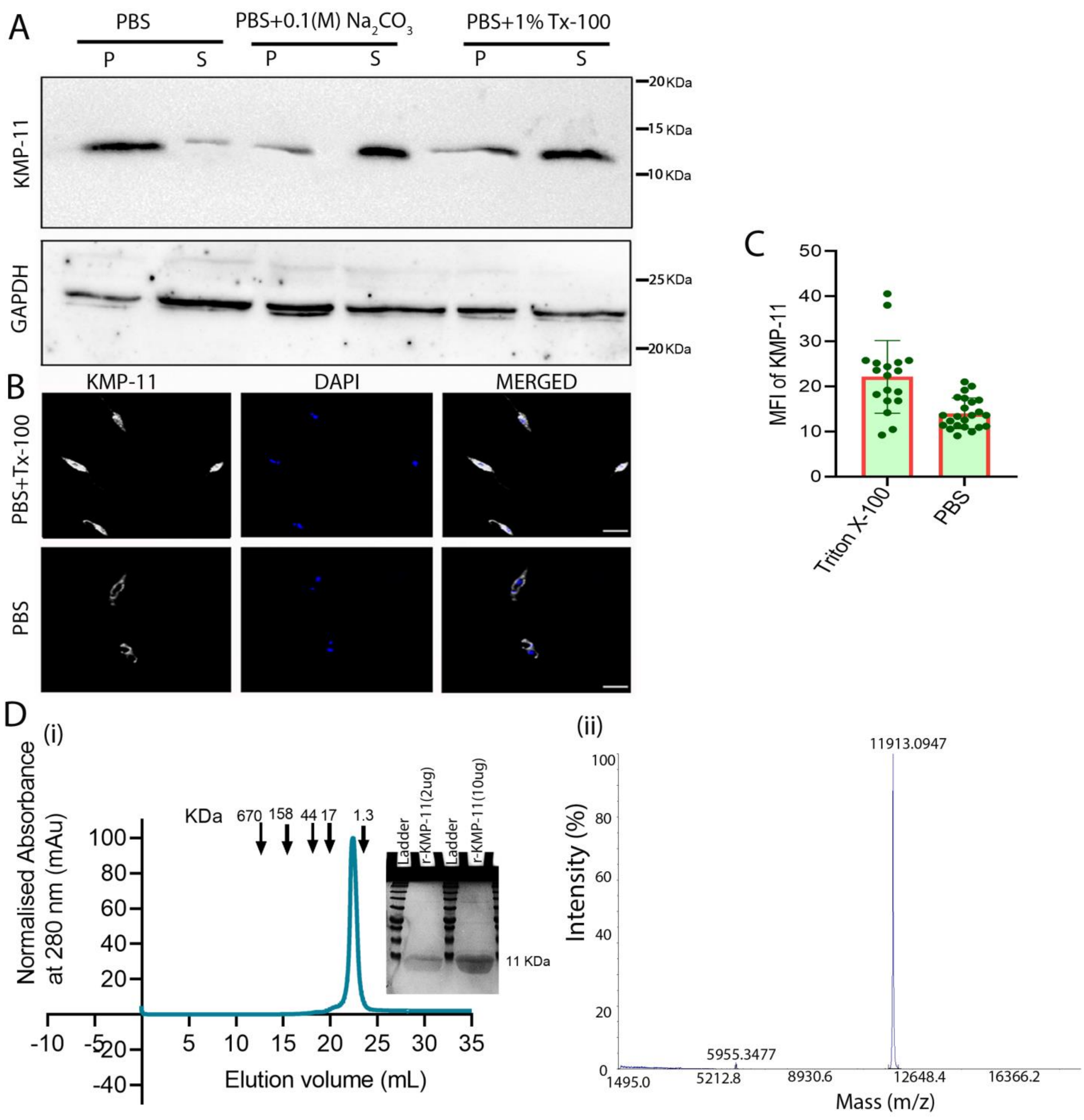

Appendix Figure S1: KMP-11 is exposed on LD surface. (A) Western Blot showing level of KMP-11 in pellet (P) and soluble fraction (S) of PBS, PBS with 0.1(M) Na_2CO_3, and PBS with 0.1% Triton X-100. It shows that KMP-11 accumulates in soluble fractions in the presence of detergent. (B) Confocal microscopy showing KMP-11 in the surface, flagella, and cytoplasm of LD in case of permeabilization with 0.1% Triton, while in absence of permeabilization, it is retained on LD surface. Scale bar is 5 μm. (C) Plot of mean fluorescence intensity (MFI) of KMP-11 on LD surface in PBS and in PBS with 0.1% TX-100. This data shows that a significant amount of KMP-11 is accessible in the LD even without permeabilization. MFI has been

calculated by selecting LDs (n=17 for PBS with 0.1%TX-100 condition and n=23 for PBS condition) from the confocal microscopic images of three biological replicates for each condition. Fiji Image J was used to evaluate the intensity. (D). (i) Gel filtration chromatography. Absorbance at 280 nm was monitored continuously to identify the purified protein. Standard molecular mass markers (thyroglobulin, 670 KDa; ϒ-globulin, 158 KDa; ovalbumin, 44 KDa; myoglobin, 17 KDa; and vitamin B12, 1.35 KDa) were chromatographed on the same column (arrows) under the same conditions. The apparent molecular mass of our purified r-KMP-11 is ~11 KDa. Inset shows the SDS page of our purified r-KMP-11. Two different amounts of r-KMP-11 (5 and 20 μg) were loaded in 15% SDS containing acrylamide gel. (ii) MALDI-TOF mass spectra of our purified r-KMP-11.

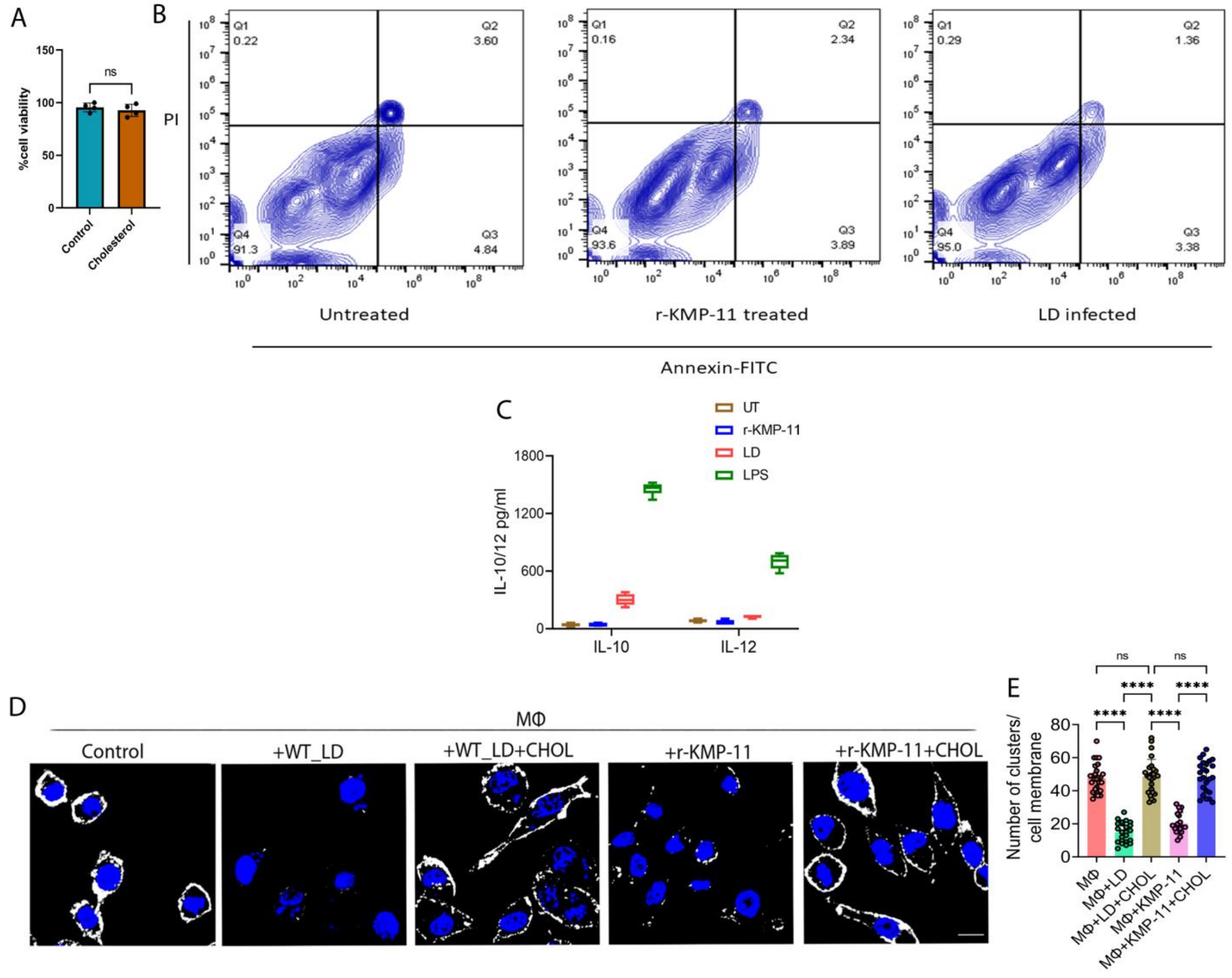

Appendix Figure S2: Effect of KMP-11. (A) MTT assay shows no toxic effect of 1.5 mM cholesterol treatment on raw 264.7 MΦ cell line. Data are represented as Mean ± SD derived

from three independent experiments. The level of significance has been estimated using unpaired t-test in GraphPad Prism (version 9) application. Here, ns p value =0.4742. (B). Flow Cytometry representing the percentage of apoptotic MΦ population in response to r-KMP-11 by Annexin V-propidium iodide (PI) staining. MΦs were treated with 50µM of r-KMP-11 for 12 hrs or left untreated or infected with LD and stained with Annexin V-FITC and propidium iodide. Percentage of apoptotic cell population was measured using flow cytometry. Data represented as contour plots and double-positive cells (Annexin V-FITC and PI) demonstrate the typical apoptotic cell population. This figure is a representative of three independent experiments. (C) Level of IL10 and IL12 in MΦs infected with LD, or treated with 50µM KMP-11, or LPS (1mg/ml) for 12 hr. Data are represented as Mean ± SD derived from two biological replicates(n=2/group). UT stands for untreated. (D). Confocal images of membrane rafts under treatment of r- KMP-11 and LD infection, and the reassembly of the raft after CHOL treatment. CTX-B-FITC was used as the raft marker. Images of control MΦ, LD-MΦ, LD- MΦ with CHOL, KMP-MΦ and KMP-MΦ with CHOL have been shown. The scale bar in each image is 10µm. To represent Ctxb clusters, all representative images for each experimental condition are uniformly represented with high contrast. (E). Plot of the number of punctate raft clusters per cell membrane for control MΦ, LD-MΦ, LD-MΦ with CHOL, KMP-MΦ and KMP-MΦ with CHOL. Data has been represented as Mean ± SD. MΦ cells (n=21) for each condition were collected from three independent confocal images and were evaluated using Fiji Image J software. The level of significance has been estimated using ANOVA in GraphPad Prism (version 9) application. Here, ns (MΦ vs MΦ+LD+CHOL) p value=0.9913 and ns (MΦ+LD+CHOL vs MΦ+KMP-11+CHOL) p value >0.9999 and ****p value <0.0001.

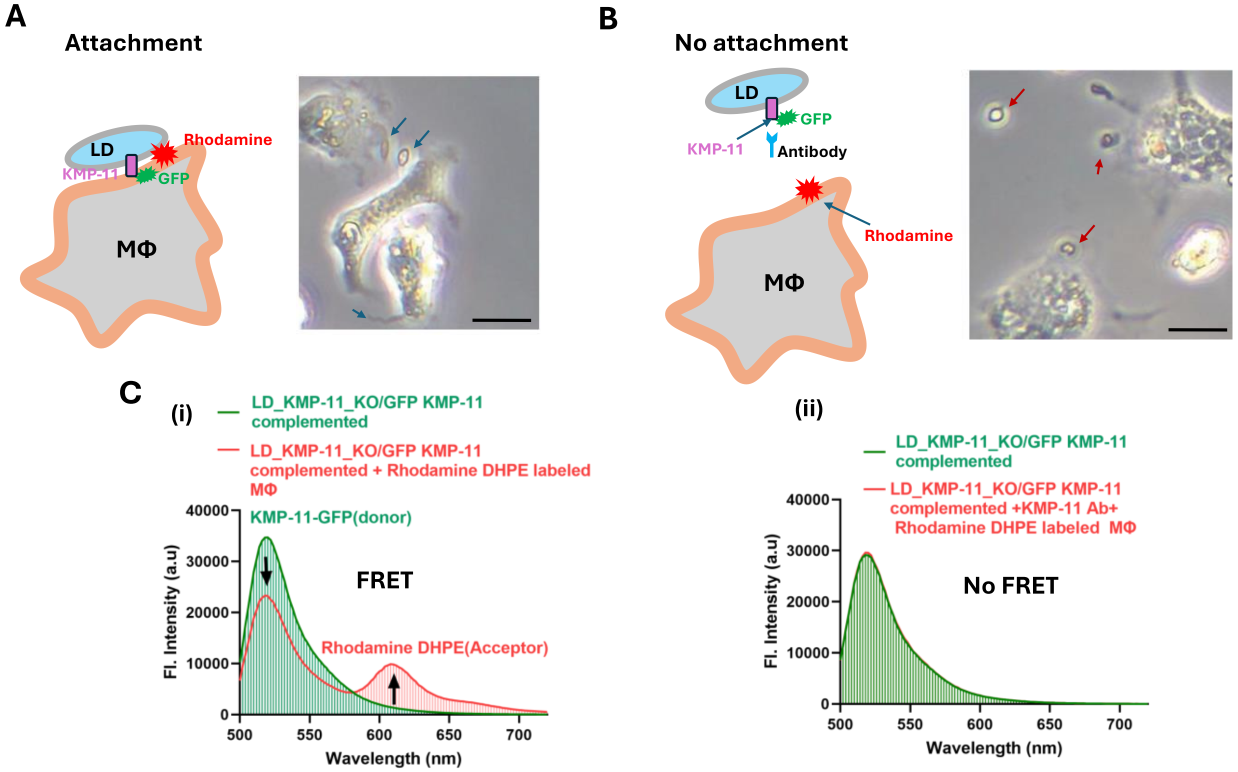

Appendix Figure S3: Model of FRET experiment to determine the binding of KMP-11 on LD with Macrophage membrane during attachment. (A.) **Condition 1:** GFP tagged KMP-11 in the KMP-11 complemented LD lines was used as donor and Rhodamine DHPE in macrophage membrane were used as acceptor. In attached condition, we can see significant FRET if donor and acceptor molecule come close to each other. Bright field image shows the attached LD (blue arrow marked) with MΦ. Scale bar is 10 μm. (B). **Condition 2:** We incubated the GFP tagged KMP-11 in the KMP-11 complemented LD lines with anti KMP-11 antibody to reduce the attachment. In unattached condition, there should not occur FRET as donor and acceptor may fall apart. Bright field image shows the unattached LD (red arrow marked) with MΦ. Scale bar is 10 μm. (C). (i) In condition 1, Fl. Intensity of GFP is reduced and an increase in Rhodamine intensity was observed thus signifying the strong FRET between KMP-11 tagged GFP and macrophage membrane bound Rhodamine. (ii). Insignificant change in emission spectra was observed in condition 2.

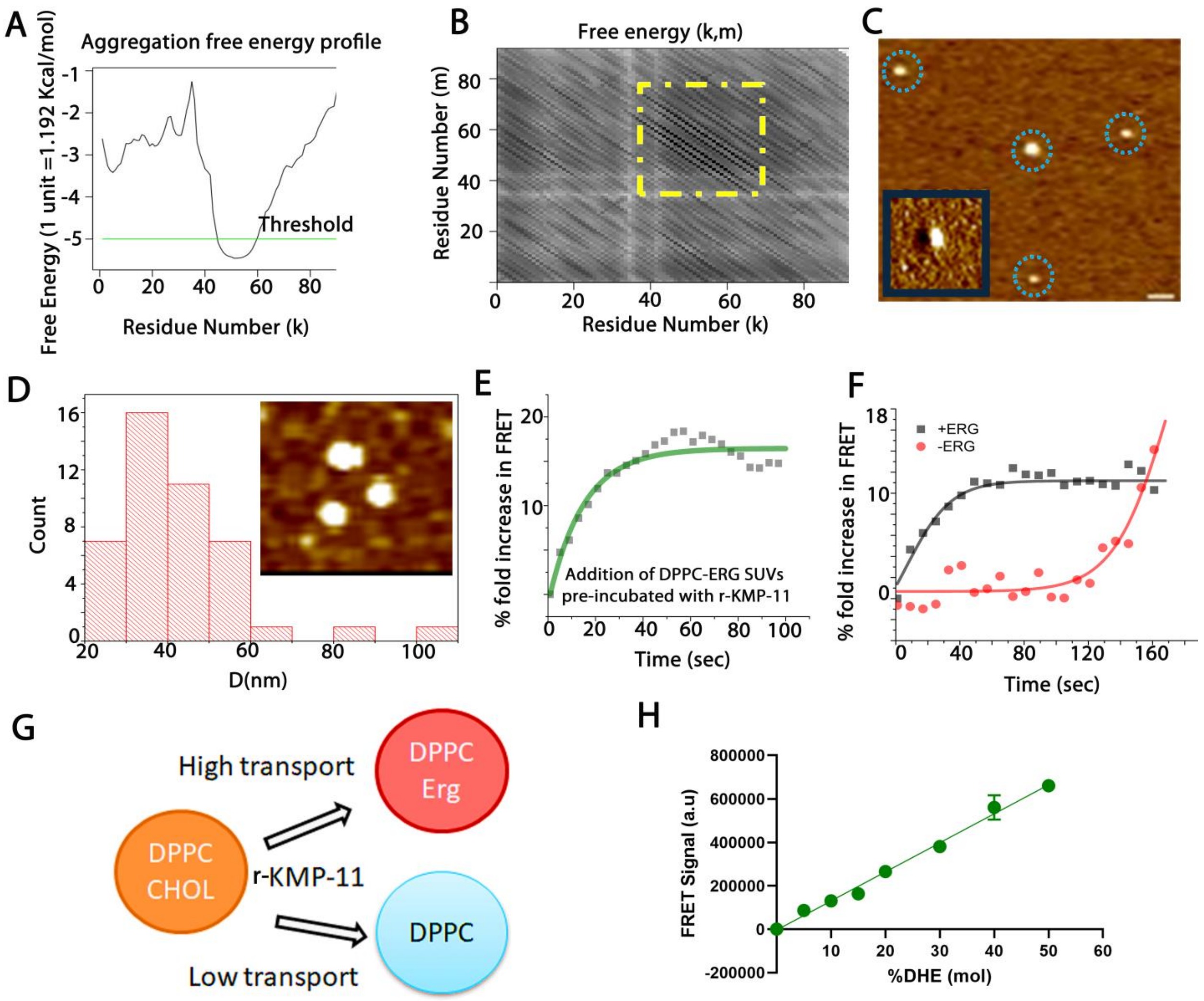

Appendix Figure S4: Oligomerization of KMP-11. (A). Using PASTA 2.0 algorithm, we calculated the aggregation/oligomerization free energy that shows 40-60 AA stretch in KMP-11 is prone to oligomer formation. (B). The free energy pairing matrix shows the oligomerization/aggregation prone region (yellow box) in KMP-11 sequence space as evaluated using PASTA 2.0 algorithm (http://protein.bio.unipd.it/pasta2/.) (C). AFM topographic image of r-KMP-11 oligomers formed in presence of membrane. Inset shows the phase topographic image of a single particle. The scale bar is 100 nm. (D). The histogram plot shows the distribution of the oligomeric particles obtained from the AFM images. The inset shows the zoomed in image of the oligomers. CHOL transport property of r-KMP-11. (E). Temporal fold increase in FRET signals. Percentage of fold increase in FRET signal between the transferred DHE (donor) and DAUDA (acceptor) after the mixing of DPPC-DHE SUVs and DPPC-DAUDA-ERG SUVs

(which were incubated with r-KMP-11 for 30 minutes). We performed this experiment to understand the mechanism of cholesterol transport. We incubated r-KMP-11 with DPPC-ERG-DAUDA SUVs assuming the oligomerization in 30 minutes window. Results suggested that the oligomers are particularly responsible for CHOL transport presumably through the hydrophobic tunneling mechanism. (F). Temporal fold increase in FRET signal for DPPC-DHE SUVs and DPPC-DAUDA SUVs (+/- ERG). Our data showed that ERG incorporation in the acceptor vesicles is showing significantly higher CHOL transport rate in comparison to the acceptor vesicles those do not contain ERG. (G). The schematic shows that KMP-11 driven cholesterol transport from DPPC-CHOL membrane is accelerated by the presence of ERG in the acceptor membrane. This data further infers that parasite membrane (that contains ERG) can facilitate the CHOL transport from the host macrophage membrane by KMP-11 during attachment. (H). FRET signal between DHE-DPPC liposome (donor) and DAUDA DPPC liposome (acceptor) in presence of r-KMP-11 (20 μM) with increasing concentration of DHE (CHOL) in donor liposomes.

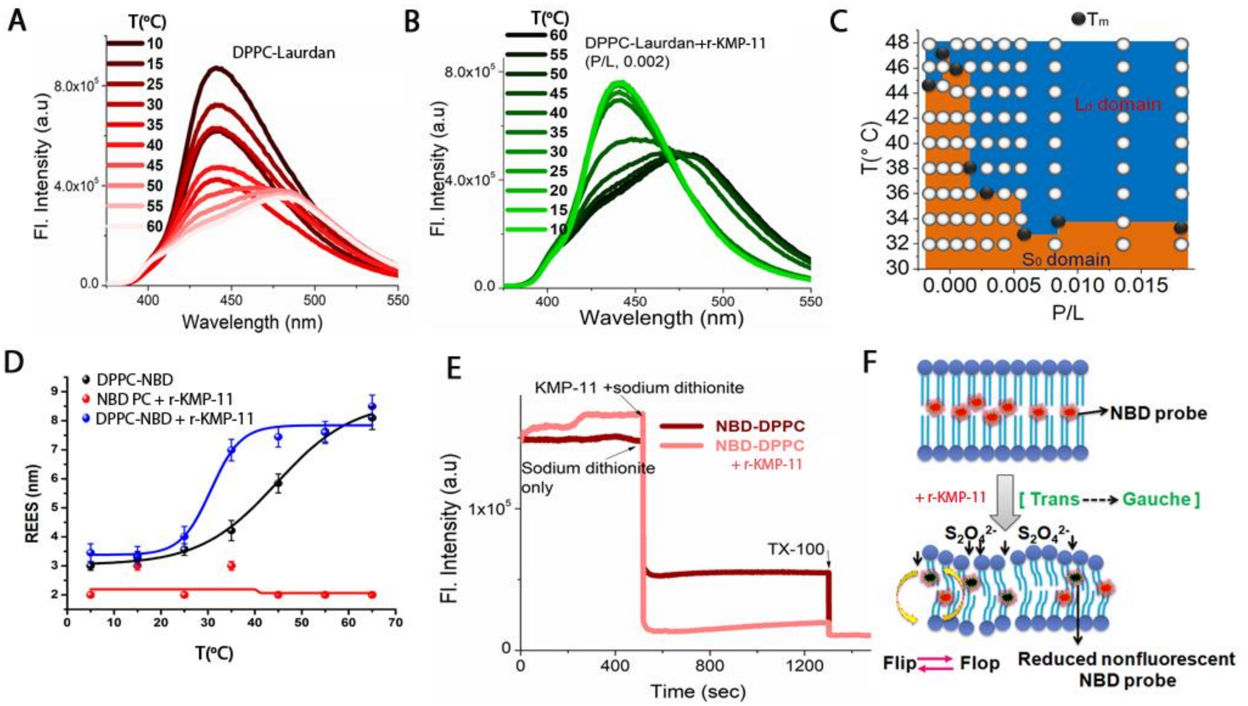

Appendix Figure S5: Measurements of the governing factors behind KMP-11 induced phase transition. DPPC laurdan fluorescence in absence and presence of r-KMP-11 with increasing temperature when P/L ratios were kept (A). 0, (B). 0.002. The color label indicates the experimental temperatures. (C). Regime diagram illustrating the changes in DPPC phase transition temperatures at different L/P ratios. (D). REES of DPPC-NBD PC and NBD-PC in absence and presence of r-KMP-11. P/L ratio was kept at 0.002 for these experiments. (E). Sodium dithionite assay for measuring the flip-flop dynamics in NBD-PC in presence and absence of KMP-11 which suggested that flip flop motion was significantly increased due to r-KMP-11 binding. (F). Schematic describes that KMP-11 increases the inner flip-flop motion in bilayer as evident from the quenching of NBD which is tagged on the nonpolar side of DPPC. This increased flip flop motion also accompanied trans to gauche conformational turn-over.

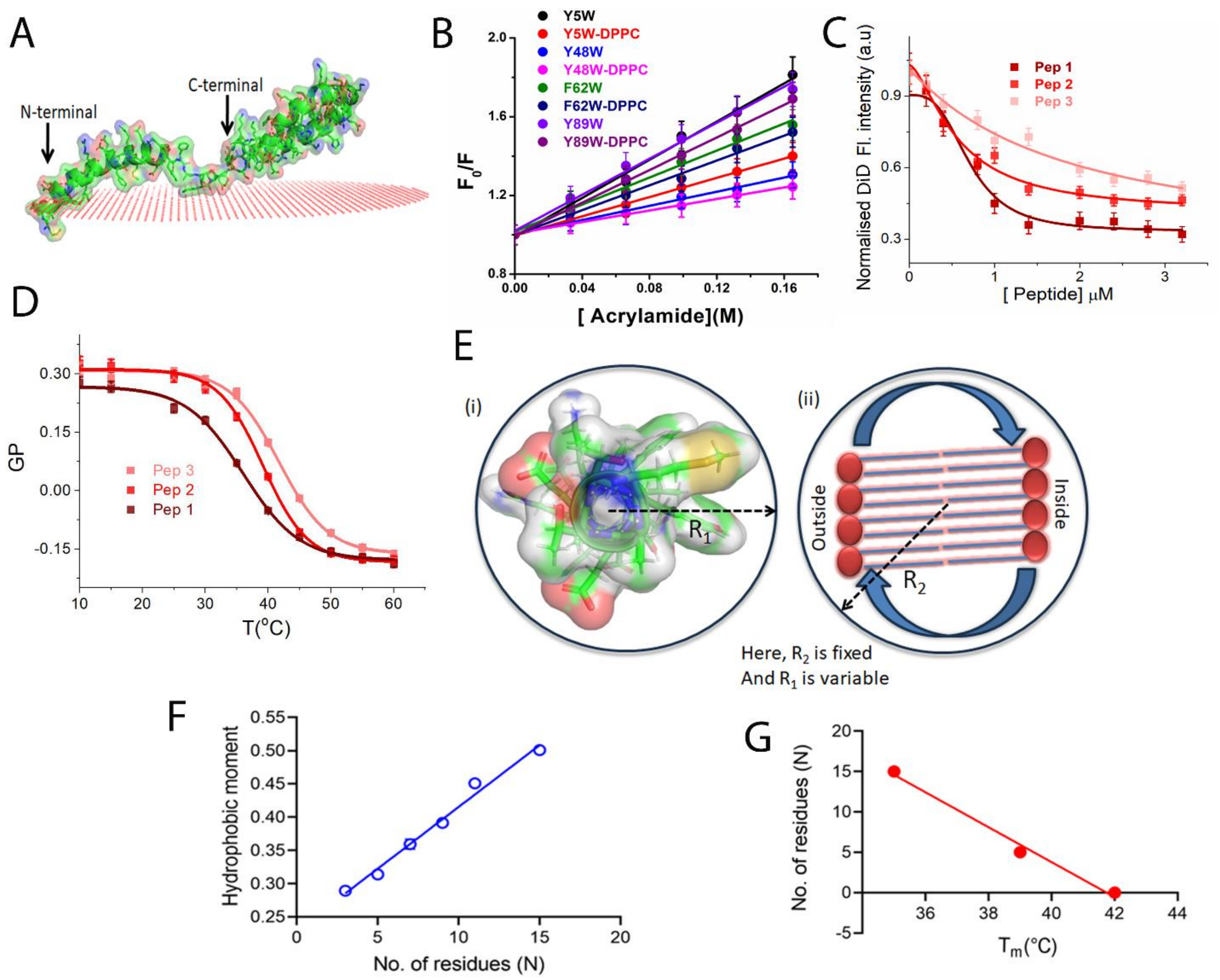

Appendix Figure S6: Amino-terminal domain is responsible for binding and subsequent changes in membrane. (A). Orientation of KMP-11 model structure in membrane as obtained from OPM computational study. (B). Representative acrylamide quenching plots (F_0/F vs acrylamide concentrations) of four mutants i.e Y5W, Y48W, F62W and Y89W in membrane bound and unbound conditions to examine the domain specific perturbations of the protein through the evaluation of stern-Volmer constants from the linear fits of the quenching data. (C). Binding of three different peptides with DPPC model membranes as monitored by the decrease in DiD fluorescence intensity with increasing concentration of added peptides. The solid lines come from the fitting of the data using Hill equation. (D). Laurdan generalized polarization of DPPC membrane in presence of different peptides. Typical peptide concentration was chosen 50 μM. Hydrophobic moment and sequence symmetry model. Two interacting domains i.e. the interacting peptide and lipid bilayer are considered spheres. (E). (i) Model Pymol structure of the amino-terminal stretch of KMP-11 (1-19AA), based on which three peptides were synthesized [peptide 1, peptide 2 and peptide 3]. Here we assume the peptide domain as a sphere model of radius R_1. (ii) On the other hand, considering the flip-flop dynamics of the bilayer entity, we assumed the specific interacting domain of the lipid bilayer as another sphere of radius R_2. R_2 is fixed since we are not changing any property of bilayer whereas R_1 is variable due to the change

in the sequence arrangement. (F). Relation between hydrophobic moment and the mirror sequence constituting residue number of the membrane interacting stretch. Linear plot of hydrophobic moment (μ_H) against the number of residues constituting the mirror sequences (N) of different combinations based on polarity and hydrophobicity of the amino acid residues in the amino-terminal domain of KMP-11. (G). Relation between mirror stretch constituting residues and chain melting temperatures of phospholipid model membranes. Linear plot of phase transition temperature (T_m) of DPPC against the number of residues constituting the mirror sequences (N) of different combinations based on polarity and hydrophobicity of the amino acid residues. The chain melting temperatures were obtained from the laurdan experiments of DPPC model membranes in presence of three synthetic peptides.

Appendix References

1. Sajadi, F. & Rowley, C.N. Simulations of lipid bilayers using the CHARMM36 force field with the TIP3P-FB and TIP4P-FB water models. *PeerJ* **6**, e5472 (2018).
2. Silverman, B.D. Hydrophobic moments of protein structures: Spatially profiling the distribution. *Proceedings of the National Academy of Sciences* **98**, 4996-5001 (2001).
3. Israelachvili, J. & Pashley, R. The hydrophobic interaction is long range, decaying exponentially with distance. *Nature* **300**, 341-342 (1982).
