# Supplementary figures and images for: Leishmania protein KMP-11 modulates cholesterol transport and membrane fluidity to facilitate host cell invasion
Source: EMBO Rep. 2024 Oct 31;25(12):5561–98. doi: 10.1038/s44319-024-00302-7 (PMC11624268; doi:10.1038/s44319-024-00302-7)

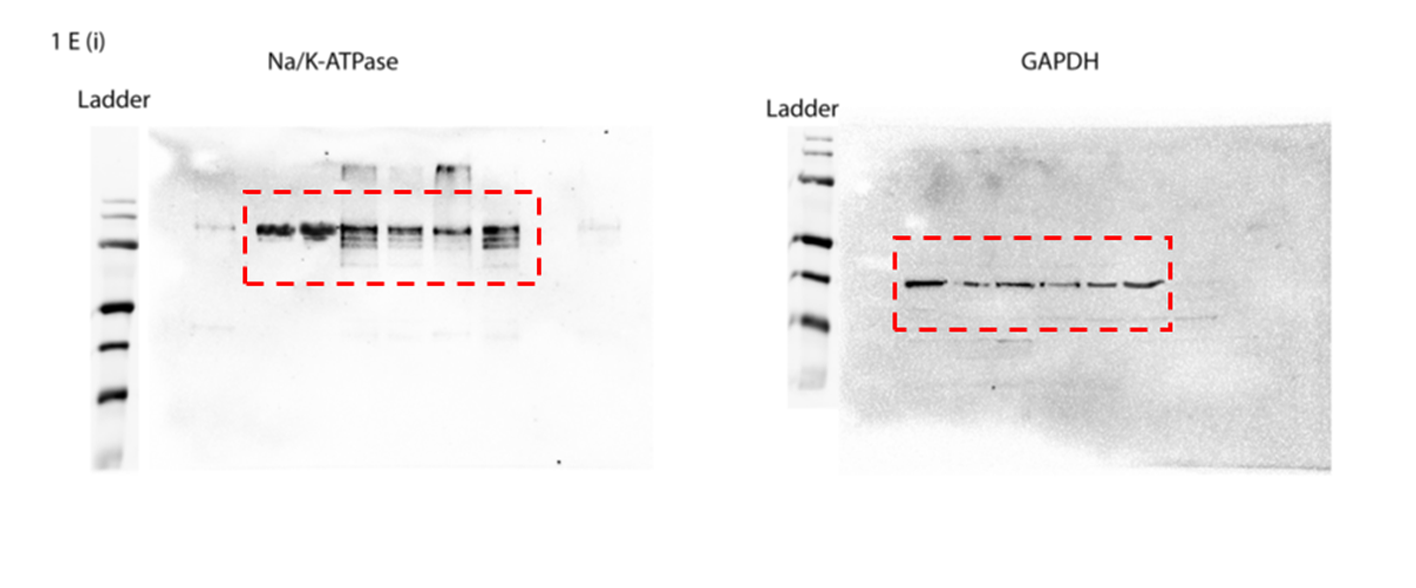

Supplement: Supplementary file 4 — Source data Fig. 1 [file 44319_2024_302_MOESM4_ESM.zip › Figure 1_SOURCE DATA/Figure 1Ei.tif]

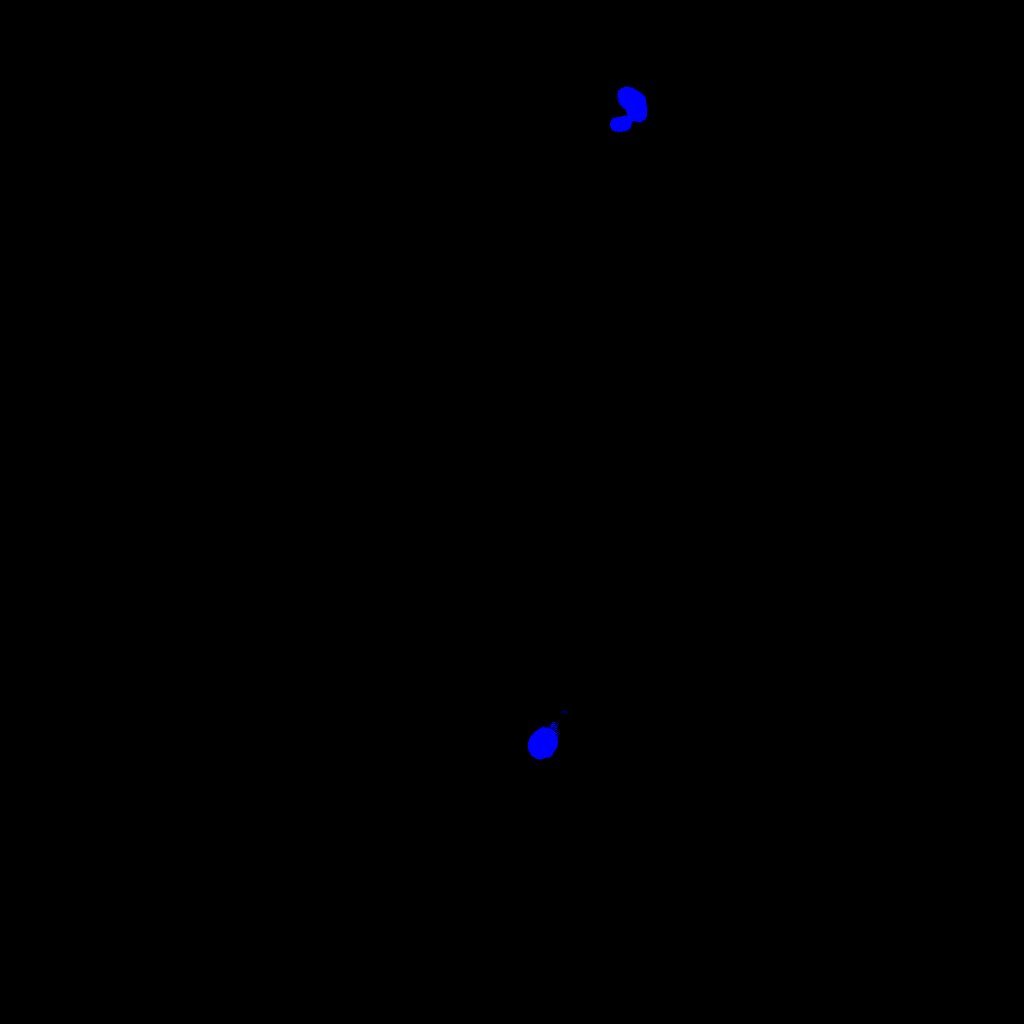

Supplement: Supplementary file 5 — Source data Fig. 2 [file 44319_2024_302_MOESM5_ESM.zip › Figure 2_SOURCE DATA/Fig. 2E/F blue.jpg (blue).jpg]

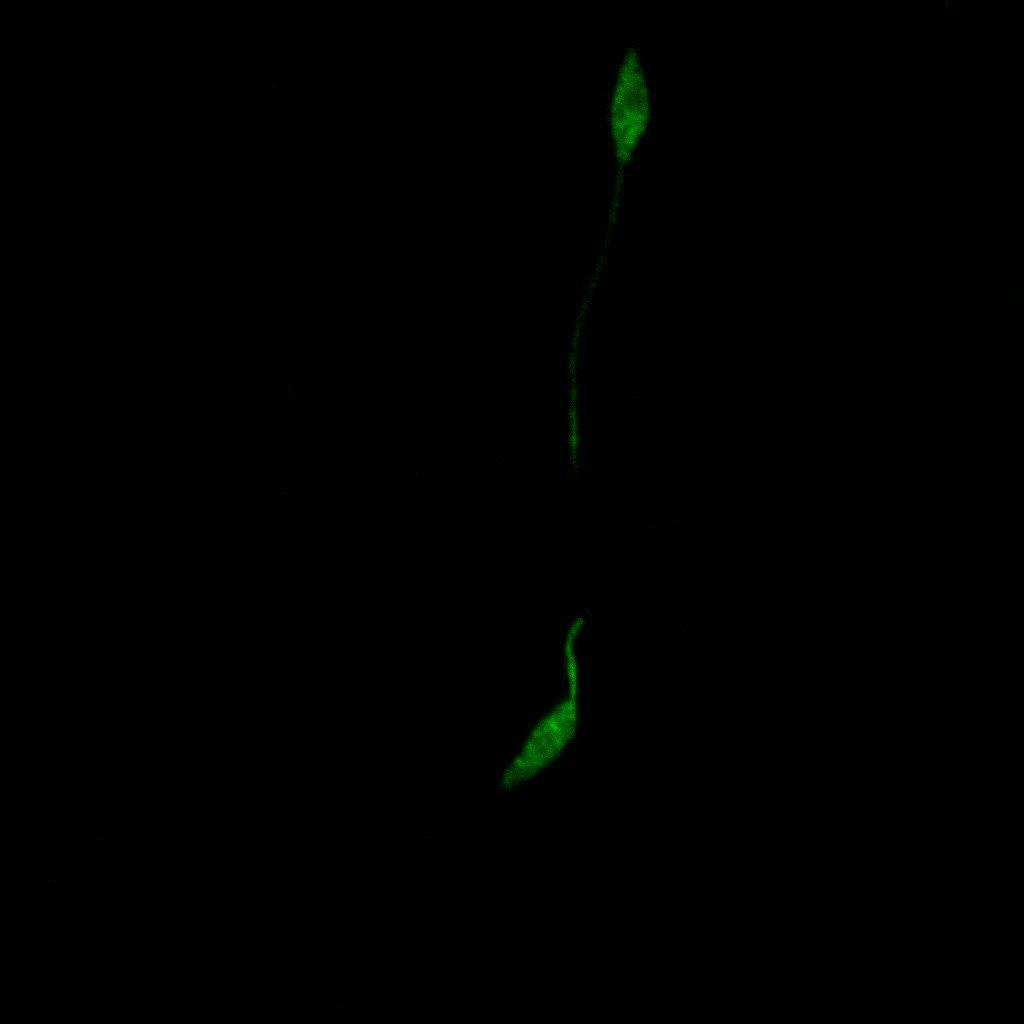

Supplement: Supplementary file 5 — Source data Fig. 2 [file 44319_2024_302_MOESM5_ESM.zip › Figure 2_SOURCE DATA/Fig. 2E/F green .jpg (green).jpg]

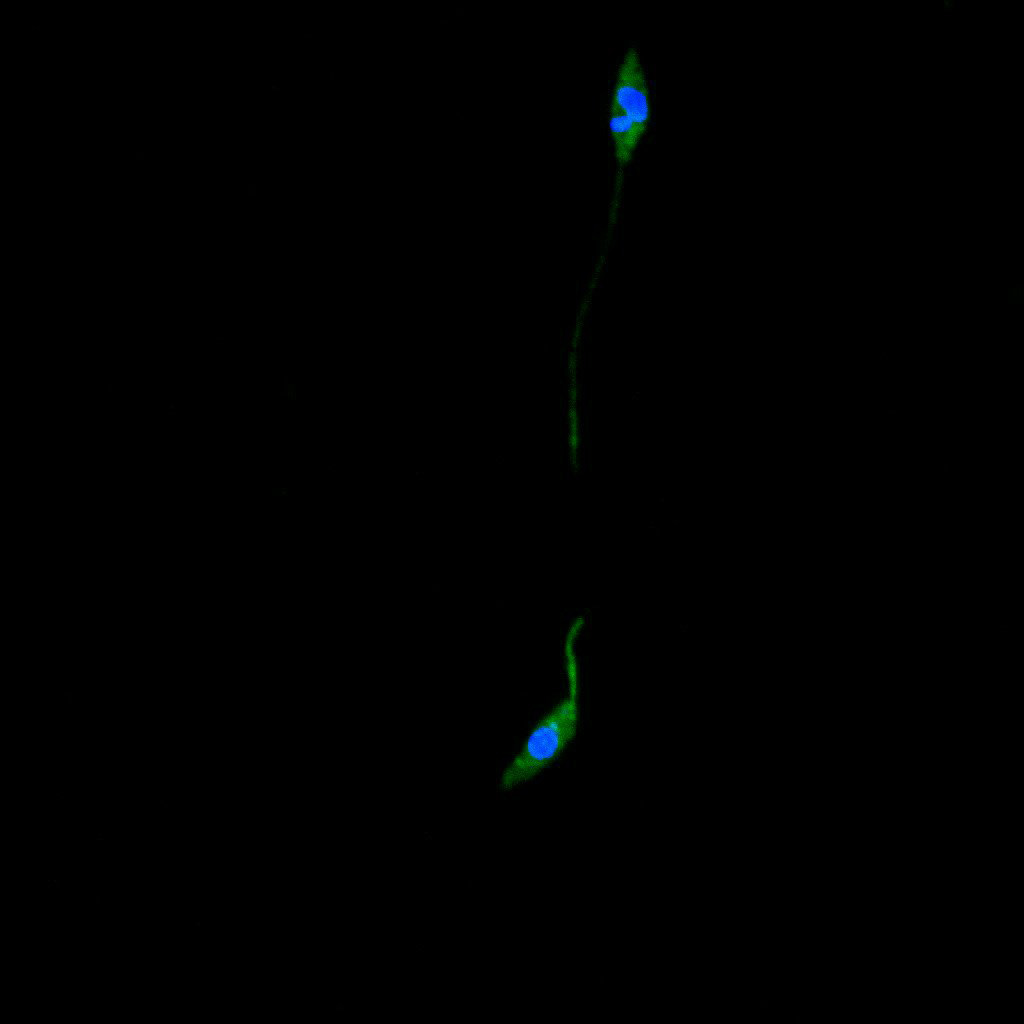

Supplement: Supplementary file 5 — Source data Fig. 2 [file 44319_2024_302_MOESM5_ESM.zip › Figure 2_SOURCE DATA/Fig. 2E/green blue Composite.jpg]

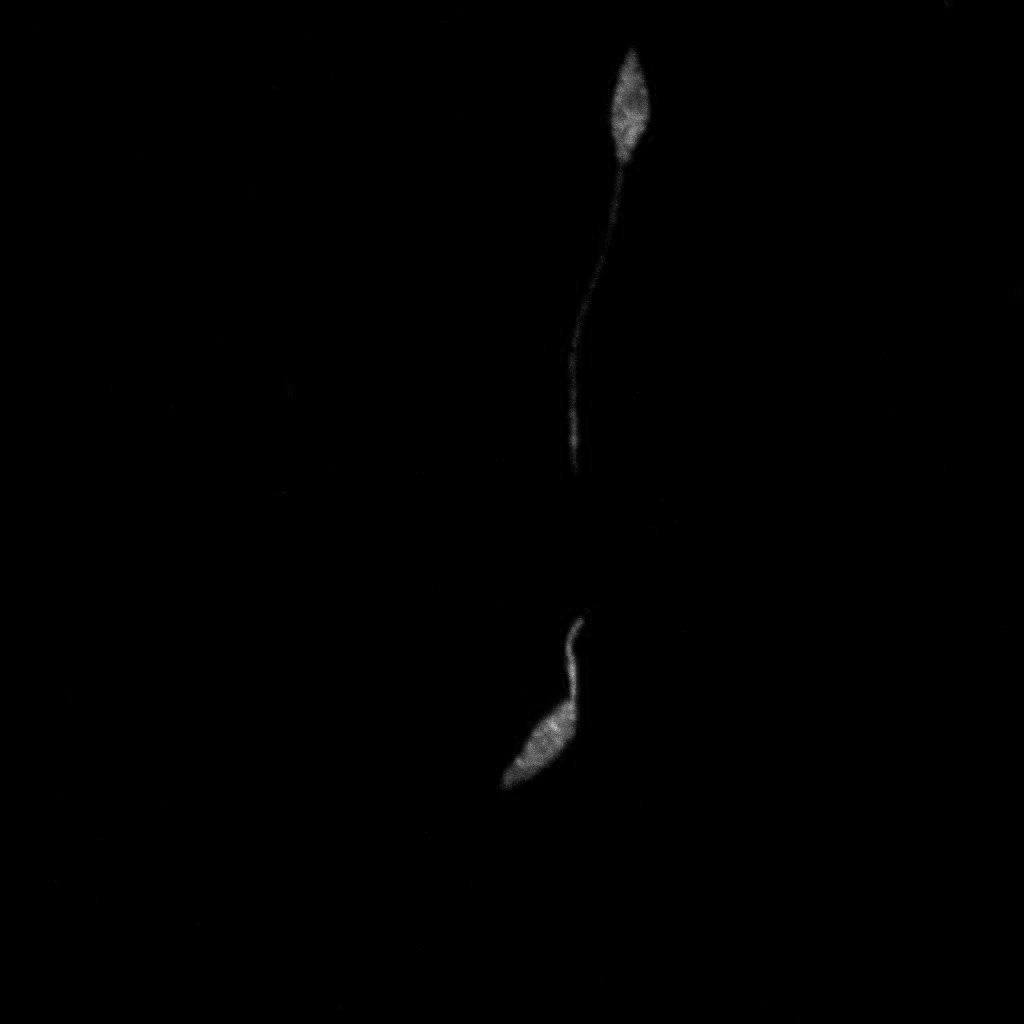

Supplement: Supplementary file 5 — Source data Fig. 2 [file 44319_2024_302_MOESM5_ESM.zip › Figure 2_SOURCE DATA/Fig. 2E/grey.jpg (green).jpg]

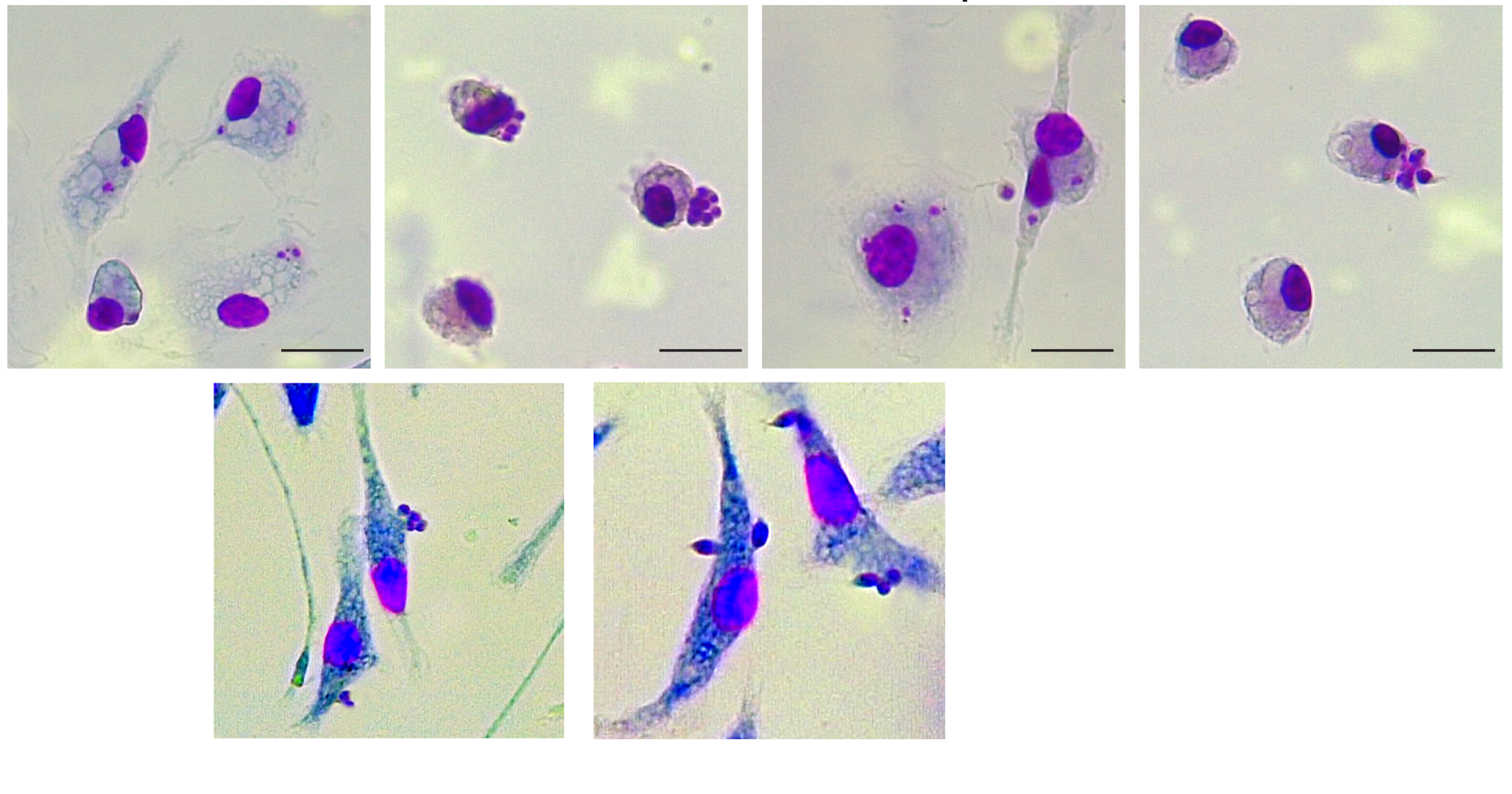

Supplement: Supplementary file 5 — Source data Fig. 2 [file 44319_2024_302_MOESM5_ESM.zip › Figure 2_SOURCE DATA/Fig. 2F.tif]

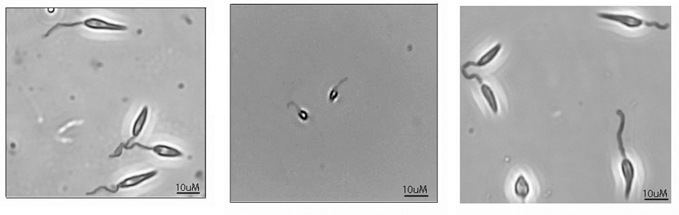

Supplement: Supplementary file 5 — Source data Fig. 2 [file 44319_2024_302_MOESM5_ESM.zip › Figure 2_SOURCE DATA/Fig.2B.tif]

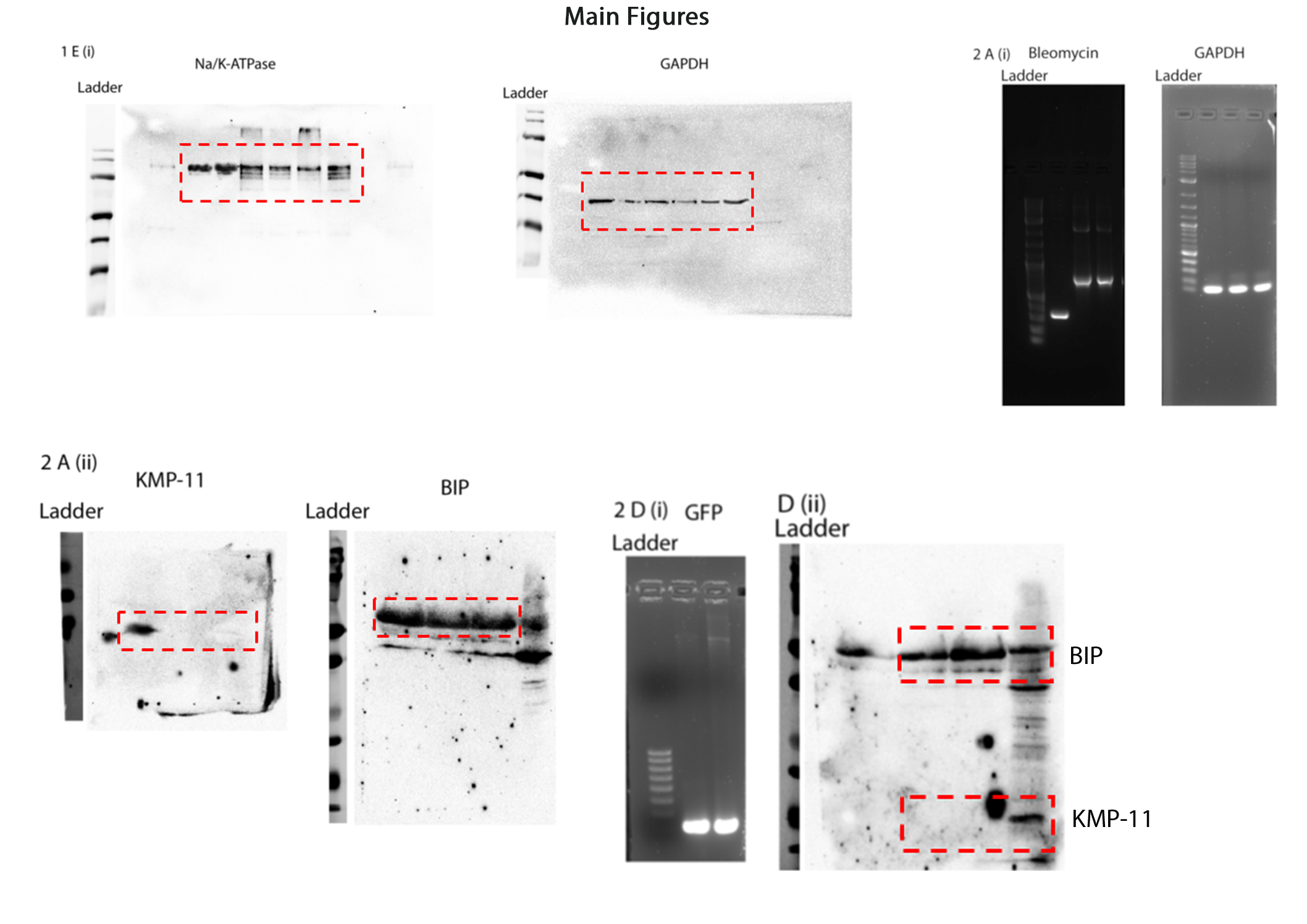

Supplement: Supplementary file 5 — Source data Fig. 2 [file 44319_2024_302_MOESM5_ESM.zip › Figure 2_SOURCE DATA/Figure 2A gel and western.tif]

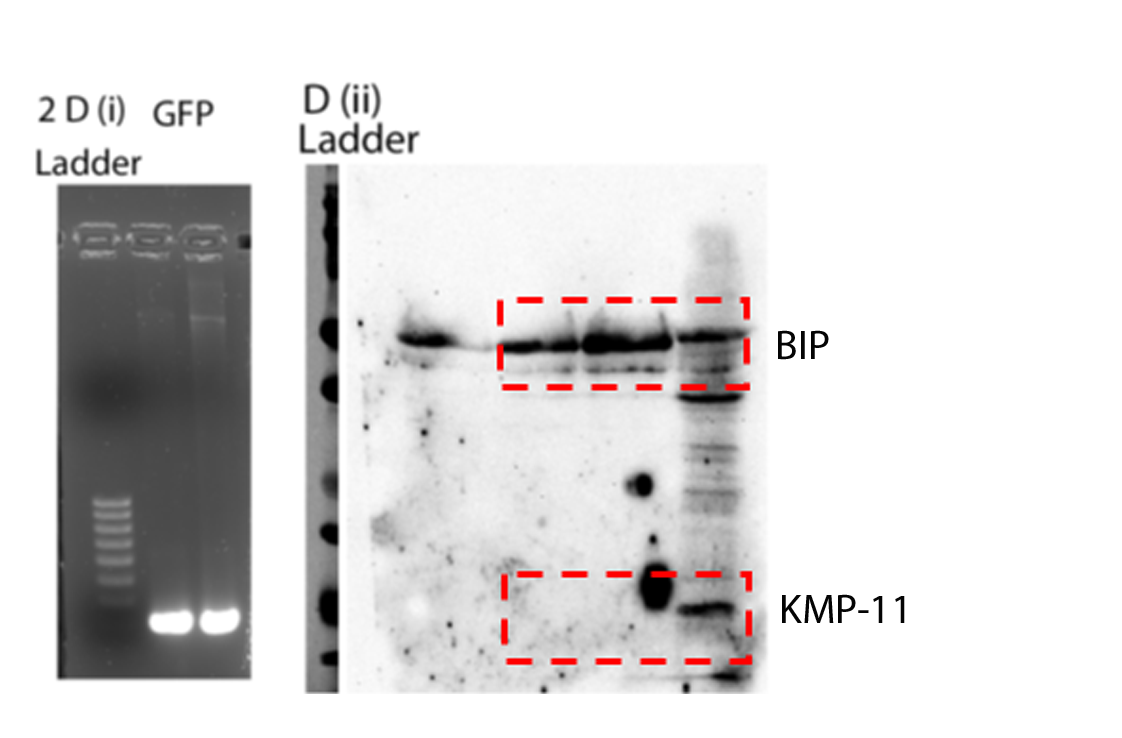

Supplement: Supplementary file 5 — Source data Fig. 2 [file 44319_2024_302_MOESM5_ESM.zip › Figure 2_SOURCE DATA/Figure 2D.tif]

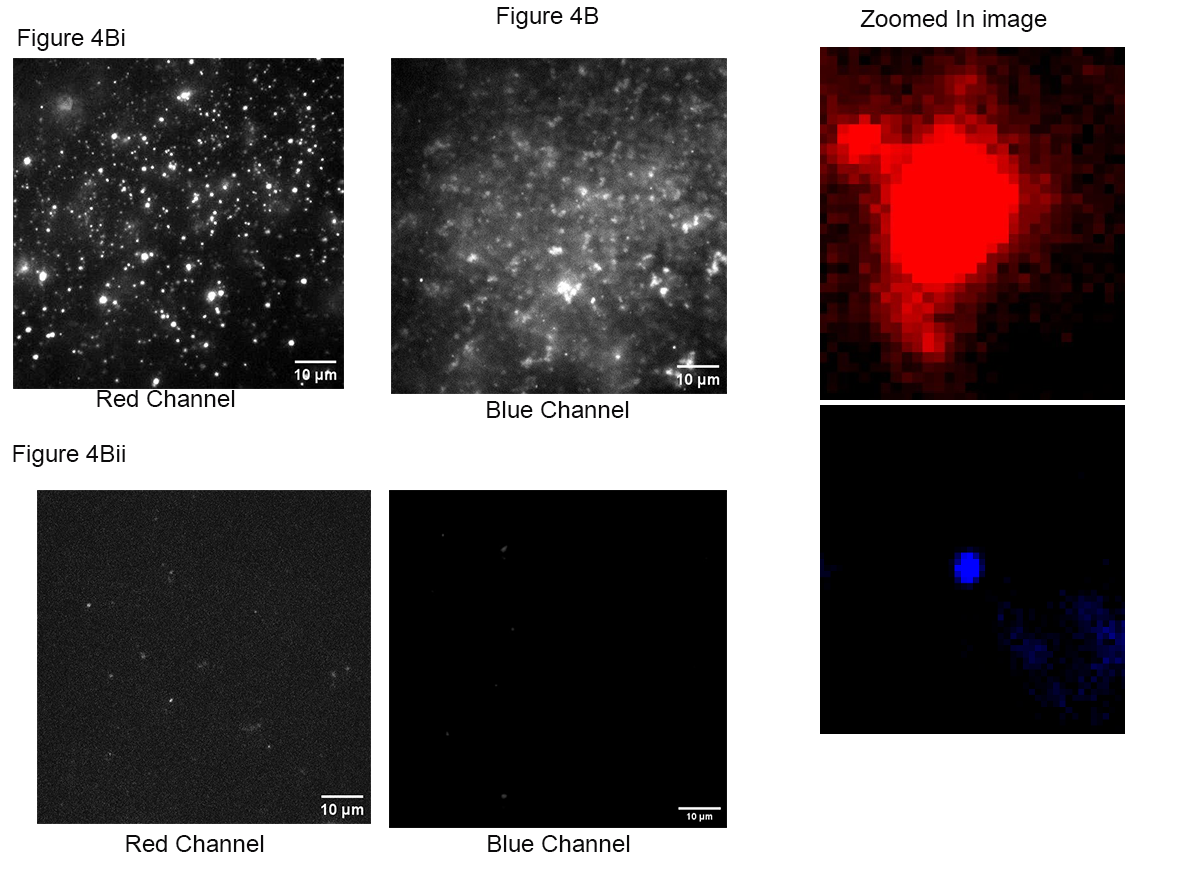

Supplement: Supplementary file 7 — Source data Fig. 4 [file 44319_2024_302_MOESM7_ESM.zip › Figure 4_SOURCE DATA/Fig. 4B.tif]

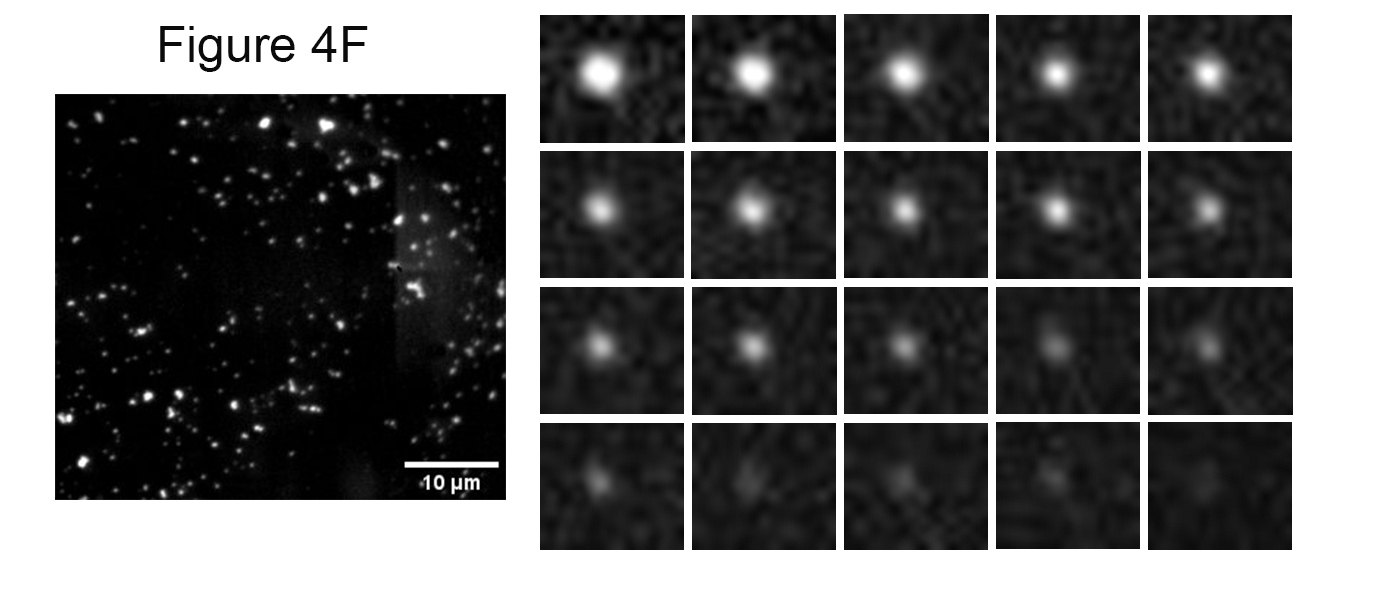

Supplement: Supplementary file 7 — Source data Fig. 4 [file 44319_2024_302_MOESM7_ESM.zip › Figure 4_SOURCE DATA/Fig. 4F.tif]

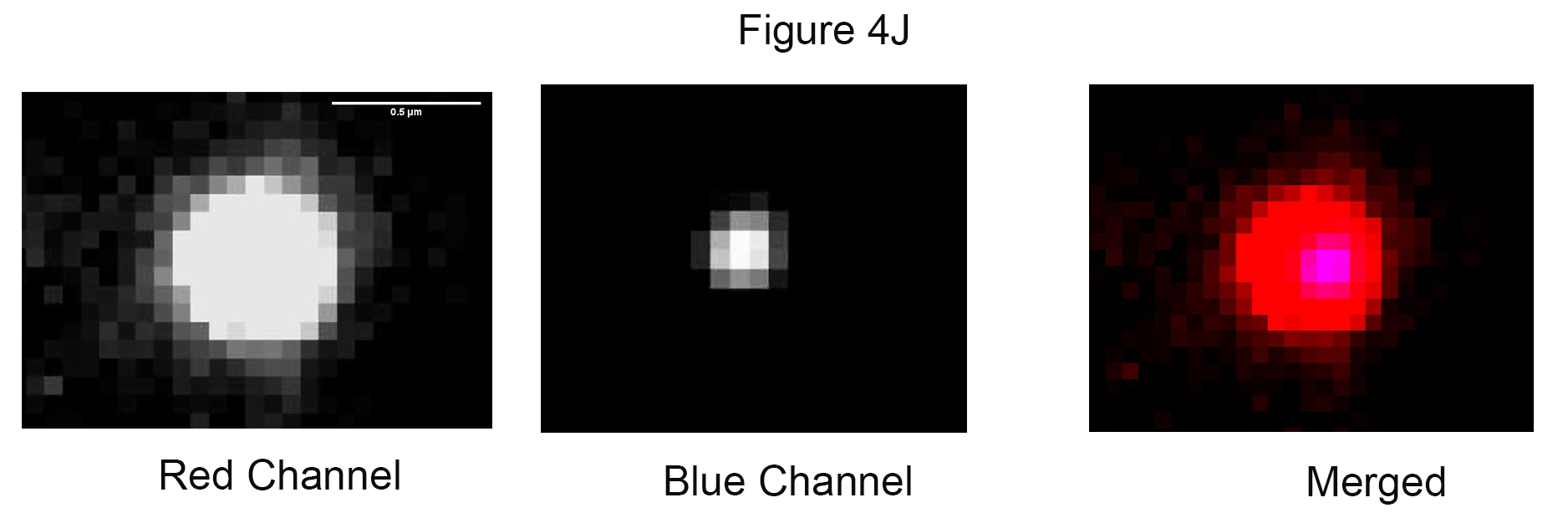

Supplement: Supplementary file 7 — Source data Fig. 4 [file 44319_2024_302_MOESM7_ESM.zip › Figure 4_SOURCE DATA/Fig. 4J.tif]

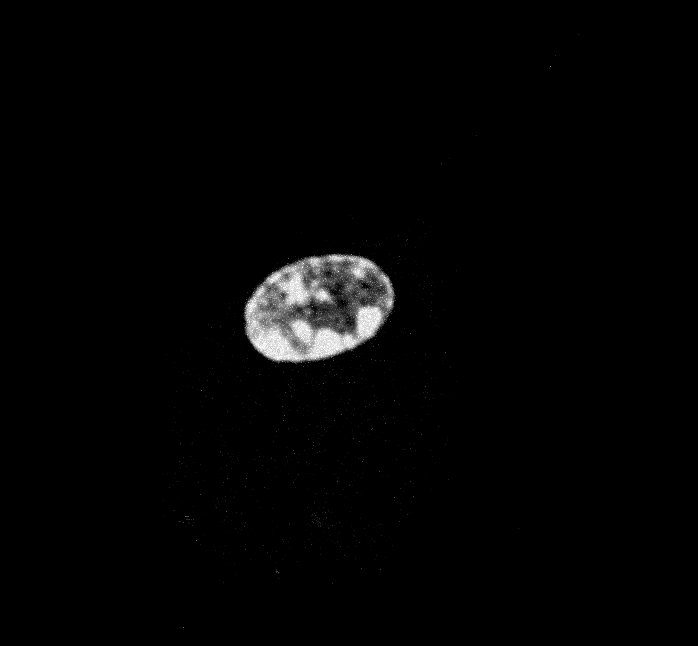

Supplement: Supplementary file 8 — Source data Fig. 5 [file 44319_2024_302_MOESM8_ESM.zip › Figure 5_SOURCE DATA/Fig. 5F/1 blue.jpg (blue).jpg]

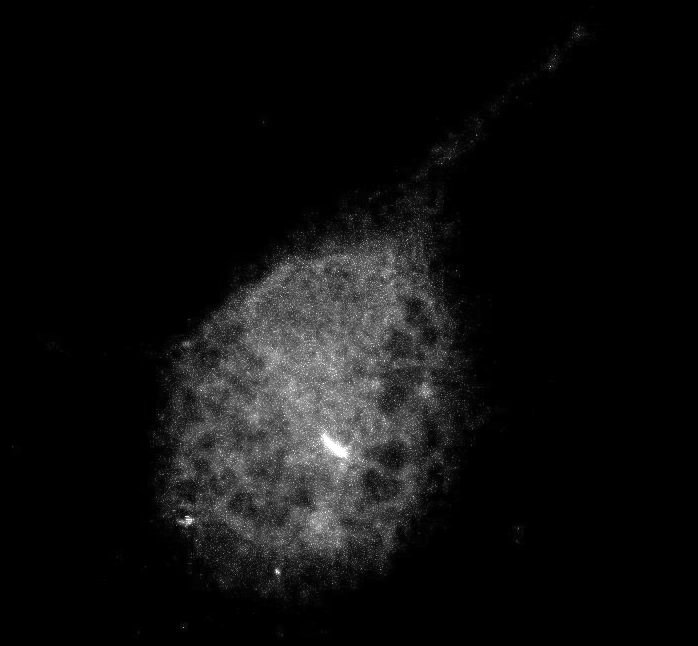

Supplement: Supplementary file 8 — Source data Fig. 5 [file 44319_2024_302_MOESM8_ESM.zip › Figure 5_SOURCE DATA/Fig. 5F/1 green.jpg (green).jpg]

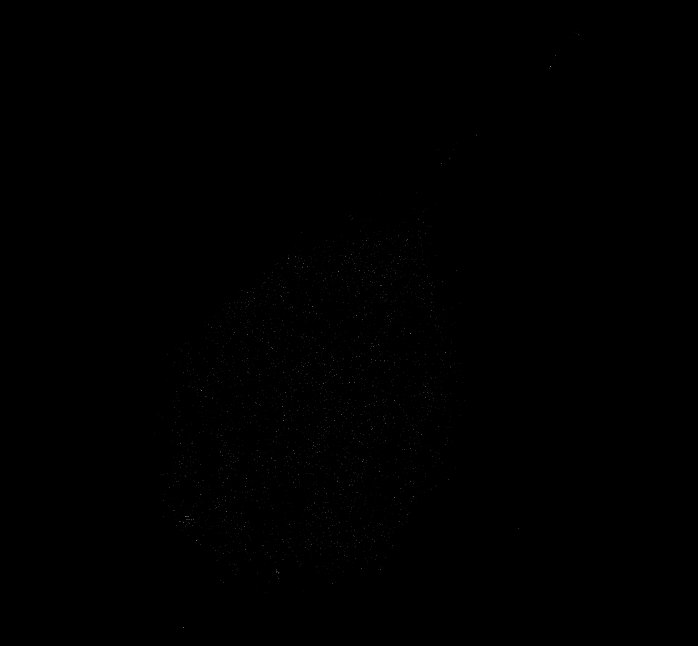

Supplement: Supplementary file 8 — Source data Fig. 5 [file 44319_2024_302_MOESM8_ESM.zip › Figure 5_SOURCE DATA/Fig. 5F/1 magenta.jpg (red).jpg]

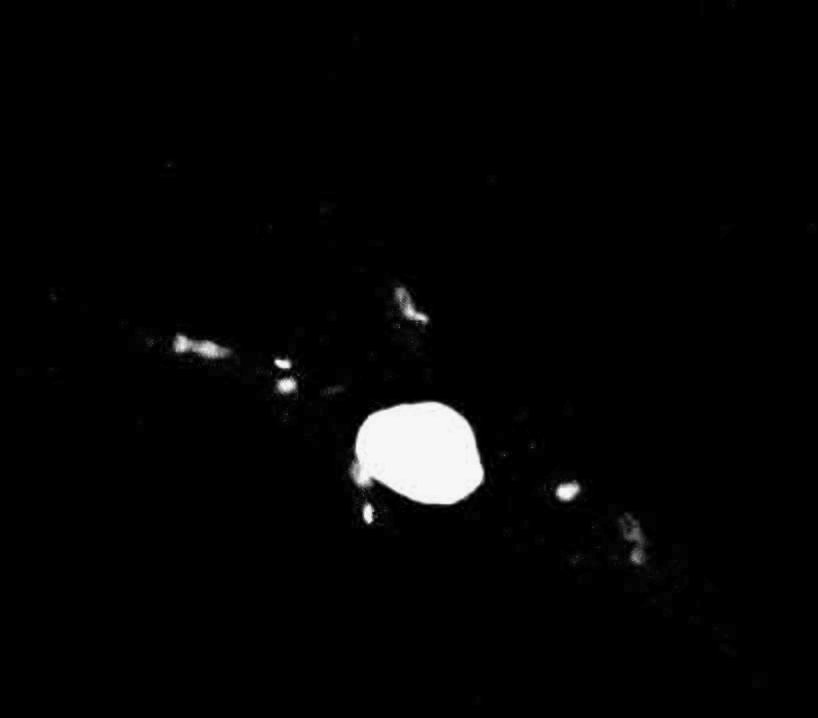

Supplement: Supplementary file 8 — Source data Fig. 5 [file 44319_2024_302_MOESM8_ESM.zip › Figure 5_SOURCE DATA/Fig. 5F/2 blue.jpg (blue).jpg]

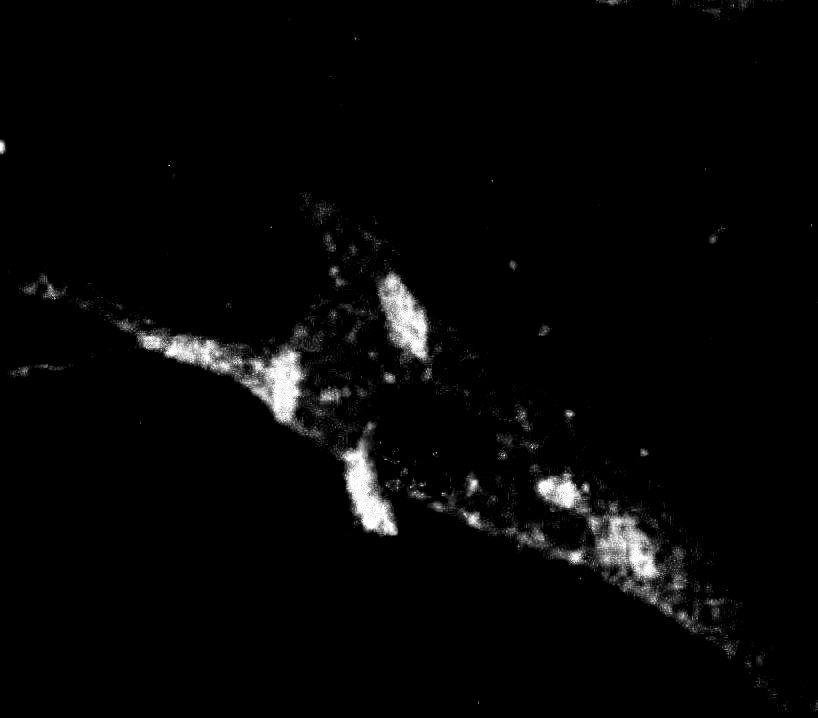

Supplement: Supplementary file 8 — Source data Fig. 5 [file 44319_2024_302_MOESM8_ESM.zip › Figure 5_SOURCE DATA/Fig. 5F/2 green.jpg (green).jpg]

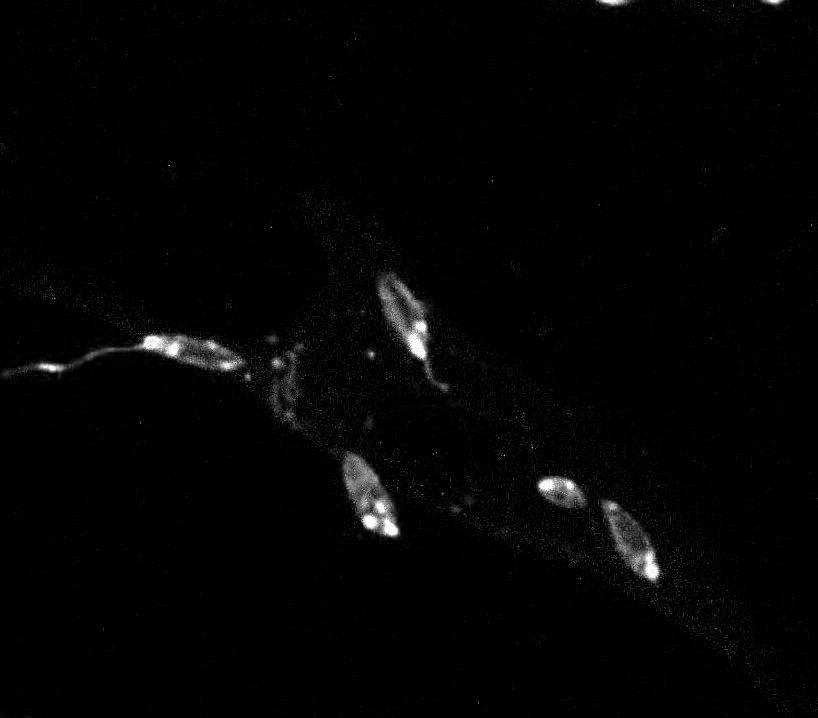

Supplement: Supplementary file 8 — Source data Fig. 5 [file 44319_2024_302_MOESM8_ESM.zip › Figure 5_SOURCE DATA/Fig. 5F/2 magenta.jpg (red).jpg]

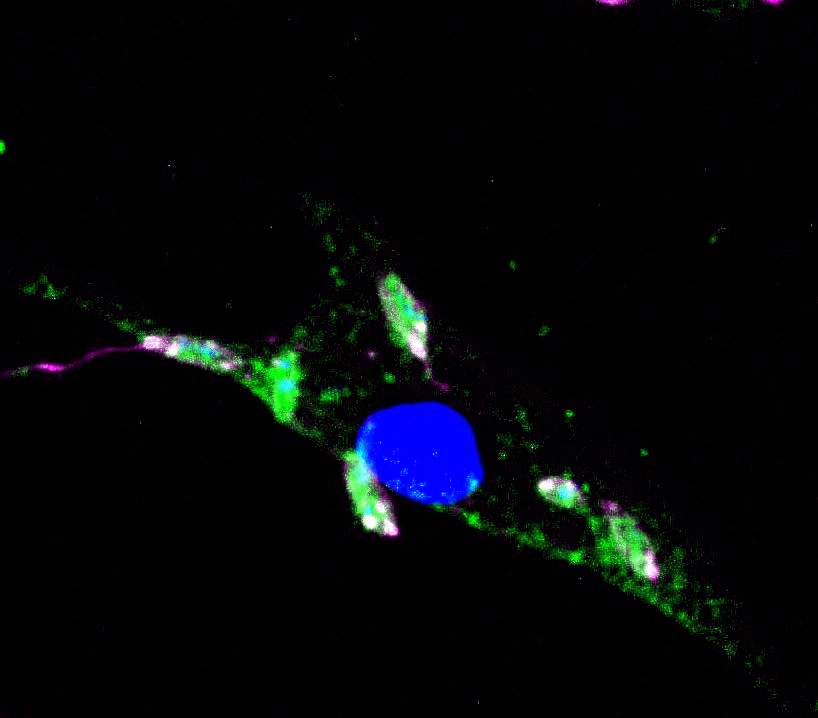

Supplement: Supplementary file 8 — Source data Fig. 5 [file 44319_2024_302_MOESM8_ESM.zip › Figure 5_SOURCE DATA/Fig. 5F/Merge Composite.jpg]

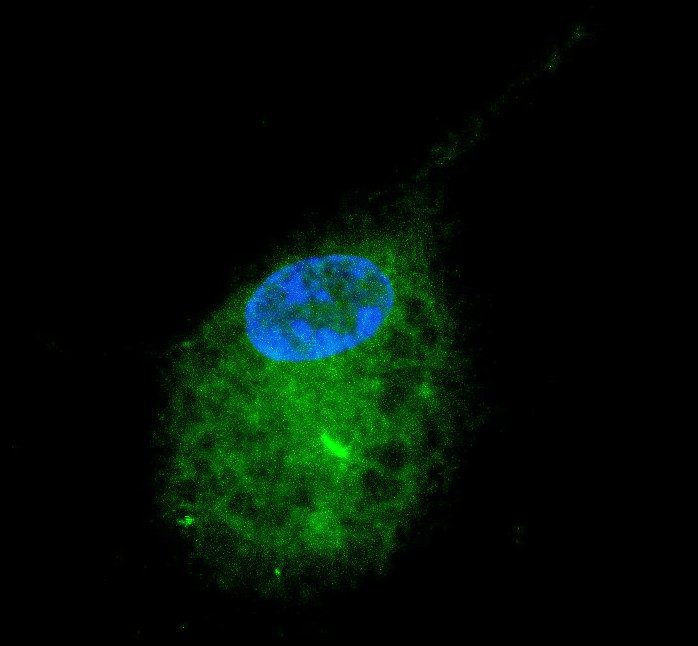

Supplement: Supplementary file 8 — Source data Fig. 5 [file 44319_2024_302_MOESM8_ESM.zip › Figure 5_SOURCE DATA/Fig. 5F/Merged all Composite.jpg]

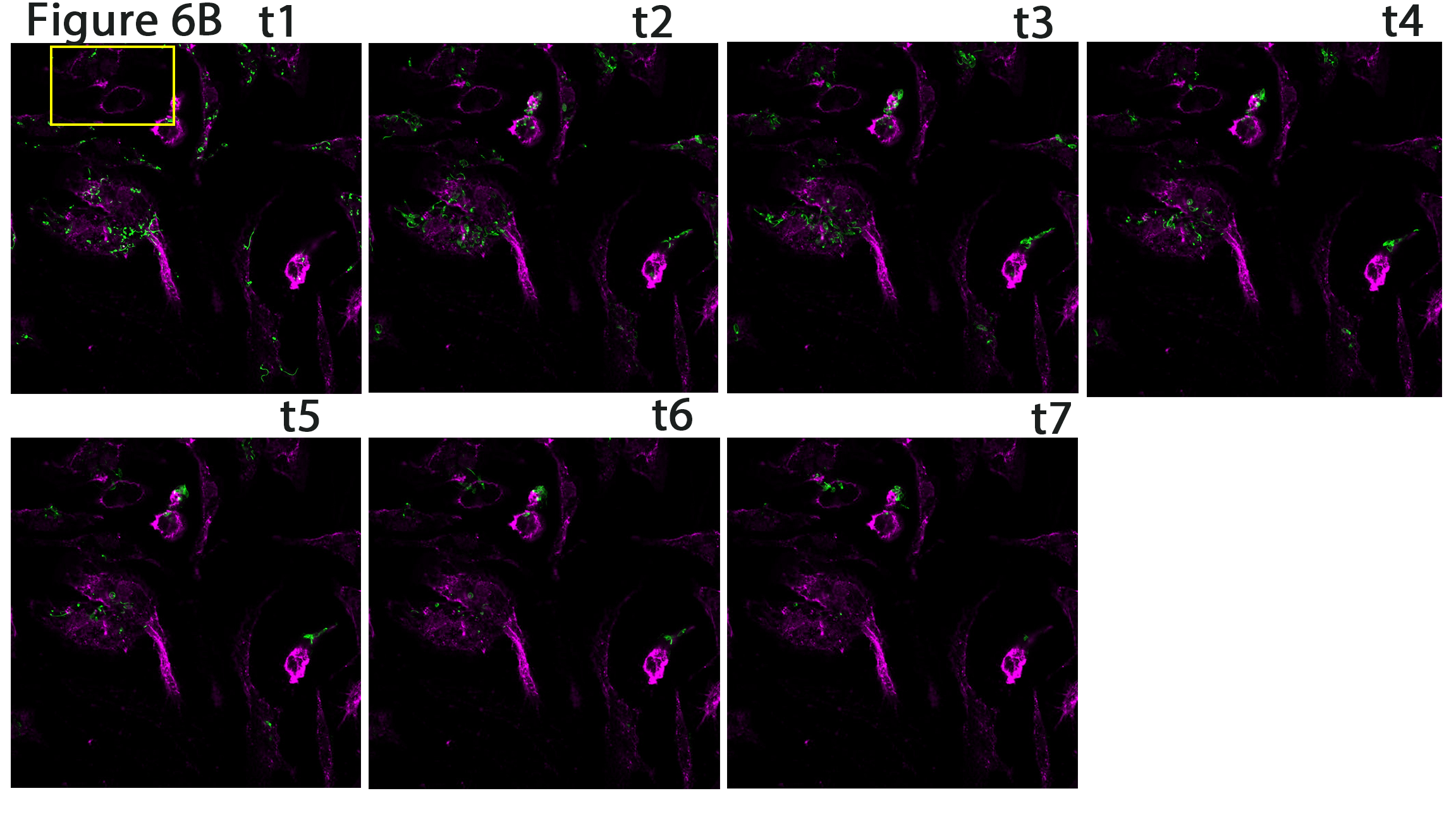

Supplement: Supplementary file 9 — Source data Fig. 6 [file 44319_2024_302_MOESM9_ESM.zip › Figure 6_SOURCE DATA/Fig. 6B.tif]

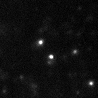

Supplement: Supplementary file 9 — Source data Fig. 6 [file 44319_2024_302_MOESM9_ESM.zip › Figure 6_SOURCE DATA/Fig.6D.tif]

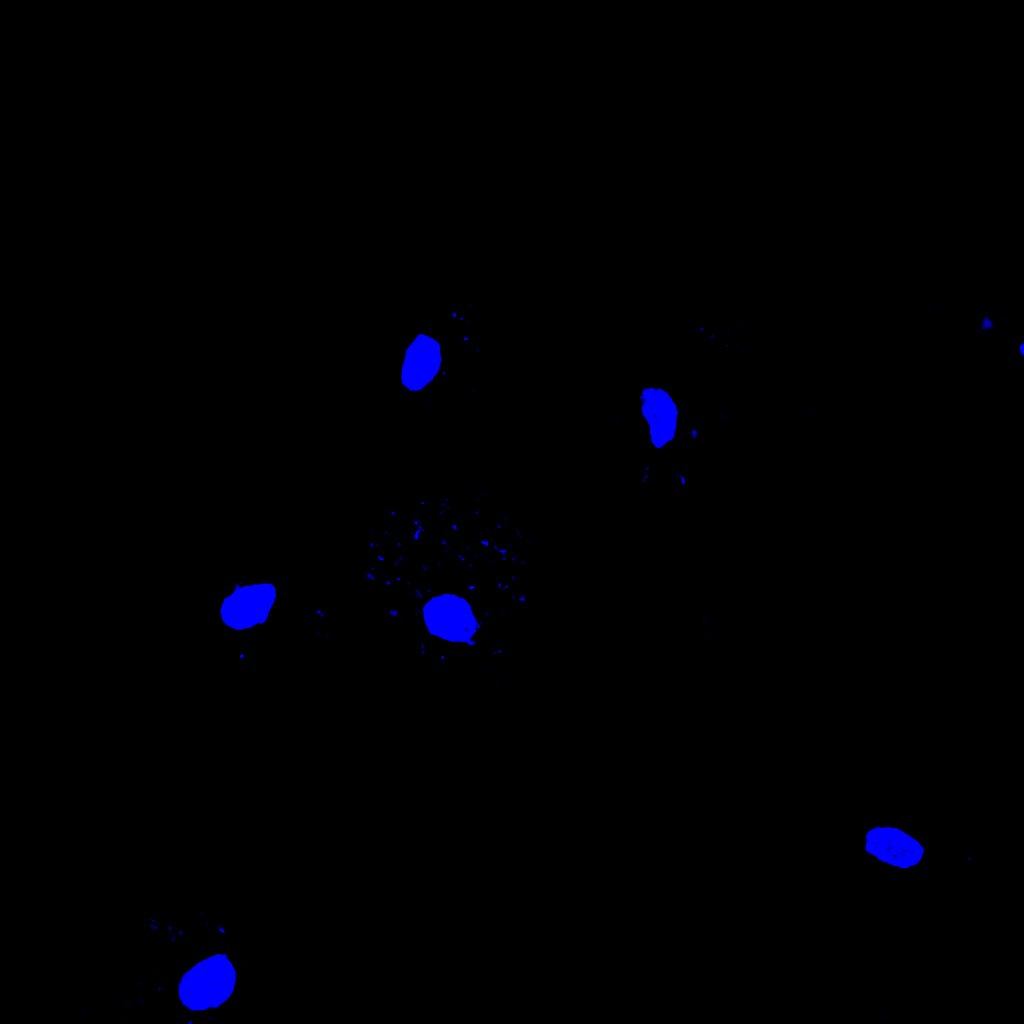

Supplement: Supplementary file 9 — Source data Fig. 6 [file 44319_2024_302_MOESM9_ESM.zip › Figure 6_SOURCE DATA/Figure 6A/1 (blue).jpg]

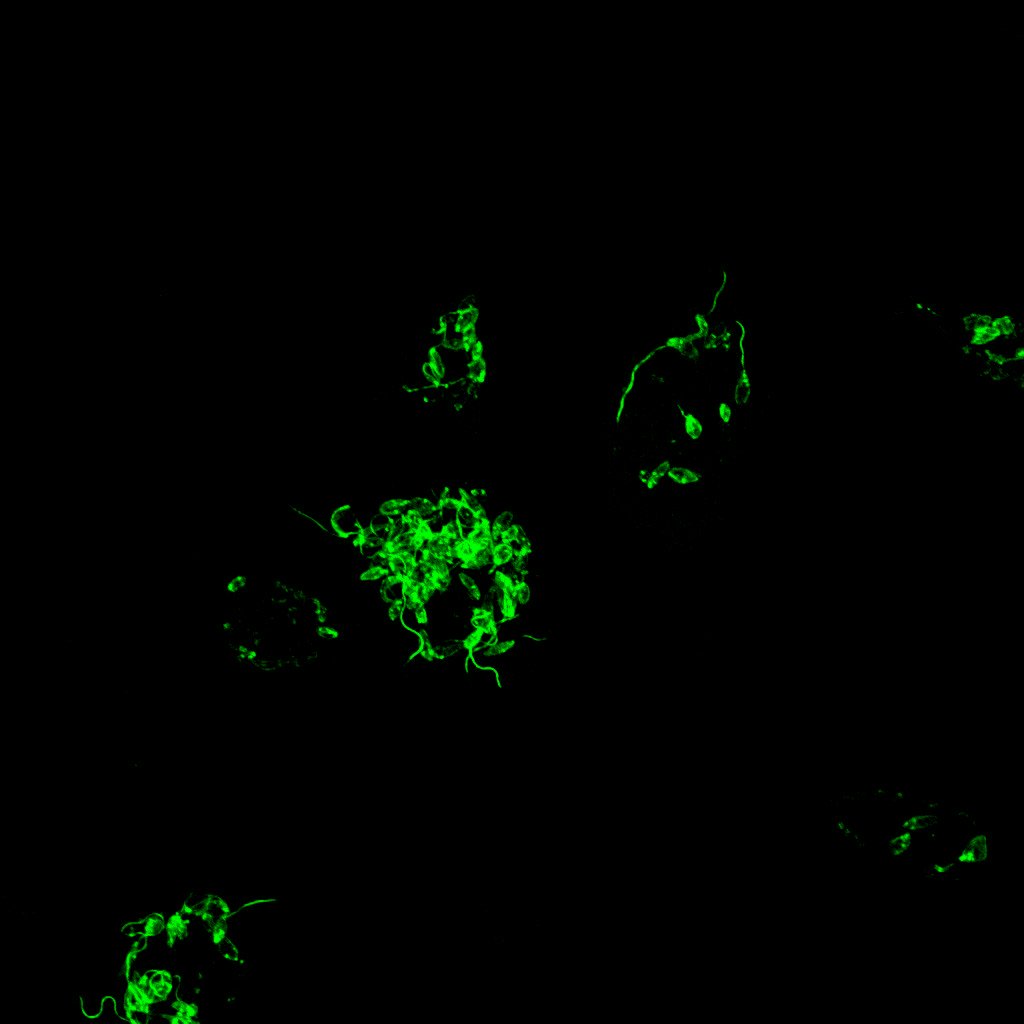

Supplement: Supplementary file 9 — Source data Fig. 6 [file 44319_2024_302_MOESM9_ESM.zip › Figure 6_SOURCE DATA/Figure 6A/1 (green).jpg]

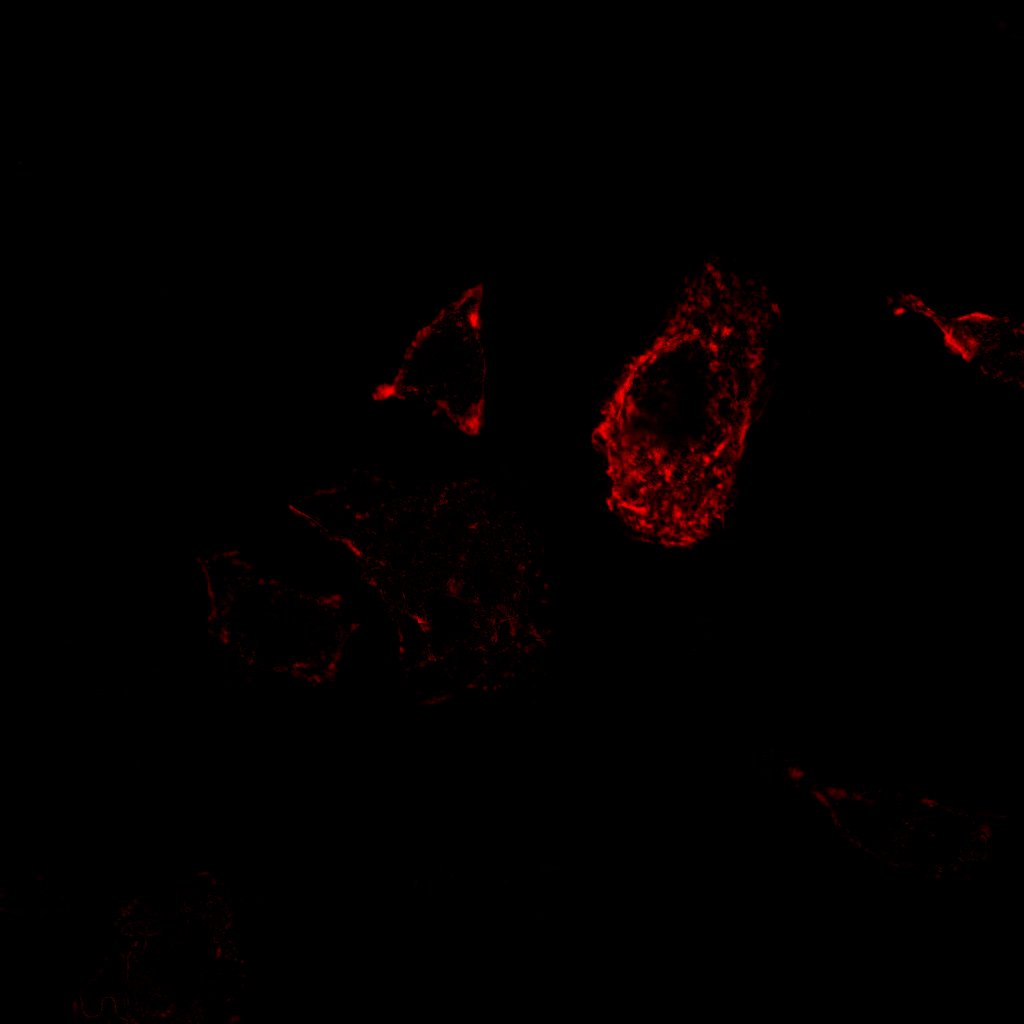

Supplement: Supplementary file 9 — Source data Fig. 6 [file 44319_2024_302_MOESM9_ESM.zip › Figure 6_SOURCE DATA/Figure 6A/1 (red).jpg]

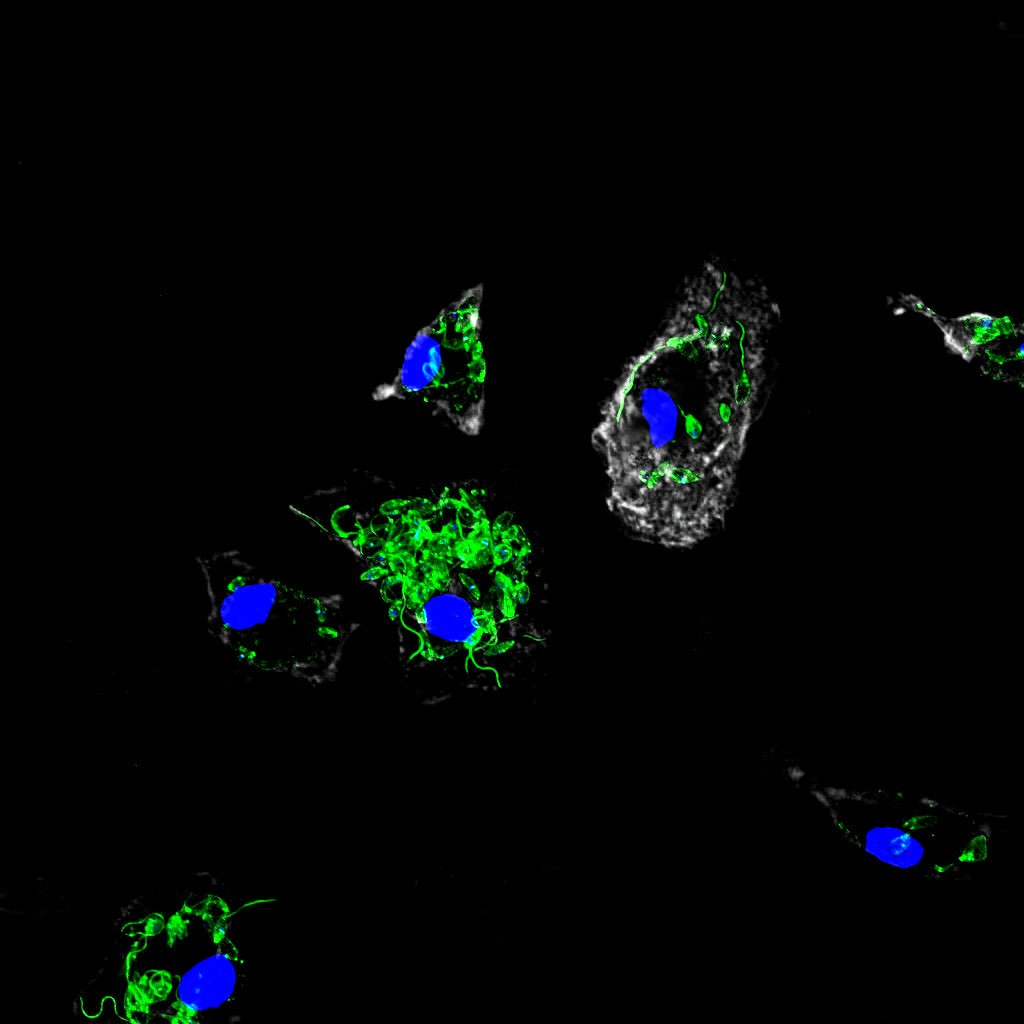

Supplement: Supplementary file 9 — Source data Fig. 6 [file 44319_2024_302_MOESM9_ESM.zip › Figure 6_SOURCE DATA/Figure 6A/F blue green grayComposite.jpg]

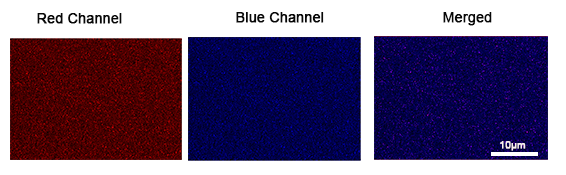

Supplement: Supplementary file 12 — Figure EV3B Source Data [file 44319_2024_302_MOESM12_ESM.zip › source data ev3b.tif]
