# Supplementary material for: Leishmania protein KMP-11 modulates cholesterol transport and membrane fluidity to facilitate host cell invasion
Source: EMBO Rep. 2024 Oct 31;25(12):5561–98. doi: 10.1038/s44319-024-00302-7 (PMC11624268; doi:10.1038/s44319-024-00302-7)
Supplement: Supplementary file 13 — Expanded View Figures [file 44319_2024_302_MOESM13_ESM.pdf]

Expanded View Figures

Figure EV1. Complementation and characterization of LD_KMP-11_KO lines.

(**A**) In vitro growth kinetics comparing WT_LD, LD_KMP11_KO and LD_KMP11_KO /KMP-11 GFP complemented lines with equal inoculums (10^2/ml) over a period of 6 days. Growth rate of WT_LD is significantly higher post 4th day of in vitro culture. The data were expressed as the mean ± SD derived from three independent experiments ($n = 3$) for each group. (**B**) MTT assay comparing viability of WT_LD,LD_KMP-11_KO and LD_KMP11_KO /KMP-11 GFP complemented lines in equal number (10^5) of WT_LD, LD_KMP-11 KO andLD_KMP11_KO /KMP-11 GFP complemented lines from a 6th day culture used for primary inoculum. Data has been presented as mean ± SD derived from three independent experiments ($n = 3$). The level of significance has been estimated using unpaired t-test in GraphPad Prism (version 9) application. Here ^{ns}P value = 0.5391. (**C**) Detection of stationary LD promastigotes by Flow cytometry on the basis of scatter light. First panel showing the WT_LD, middle panel showing the KMP-11KO LD and the right panel showing the KMP-11 complemented KMP-11 KO LD. This data is a representative of three independent experiments. (**D**) Flow cytometry representing percent metacyclic in stationary phase (6th day culture) of WT_LD, LD_KMP-11_KO and LD_KMP11_KO /KMP-11 GFP complemented lines. WT_LD lines showing slightly higher percentage of metacyclic than LD_KMP-11_KO and LD_KMP11_KO /KMP-11 GFP complemented lines. Data has been represented as mean ± SD ($n = 5$, the number of independent experiments for each group).The level of significance has been estimated using ANOVA in GraphPad Prism (version 9). Here, ^{ns}P value = 0.3382 (WT_LD vs LD-KMP-11_KO) and ^{ns}P value = 0.8312 (WT_LD vs LD-KMP-11_KO/KMP-11 complemented). (**E**) A significant higher expression of KMP-11 was observed by western blot in case of WT_LD (passage 3, P3) as compared to LD lines having more than 200 passages (LD > P200) in M199 in vitro culture. Macrophage membrane fluidity change and CHOL depletion by KMP-11. (**F**) The values of FA of MΦ membrane upon the treatment of WT_LD, LD_KMP-11_KO, LD > P200, LD_KMP-11_KO/KMP-11 GFP Complemented and LD_KMP-11_KO, LD > P200 in presence of exogenous r-KMP-11 (50 μM). Data has been represented as mean ± SD ($n = 2$, number of independent experiments). (**G**) Membrane fraction was isolated from the MΦs by ultracentrifugation. Total membrane cholesterol (free and esterified) was estimated using the Amplex-Red kit and expressed as μg of cholesterol/μg of total protein. MΦ membranes were treated with WT_LD, LD_KMP-11_KO, LD > P200, LD_KMP-11_KO/KMP-11 GFP Complemented and LD_KMP-11_KO, LD > P200 in presence of exogenous r-KMP-11 (50 μM). Data has been represented as mean ± SD ($n = 2$, number of independent experiments).

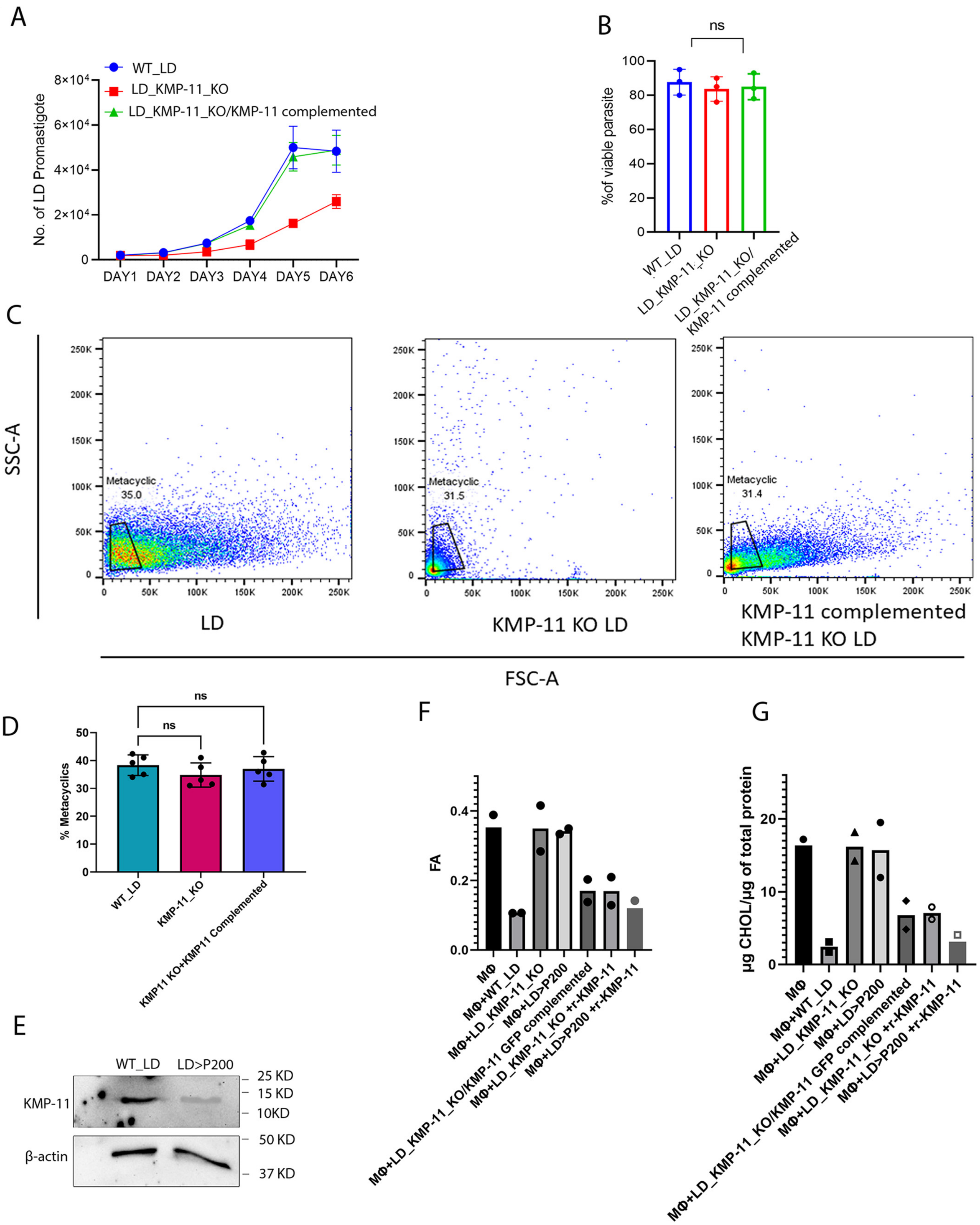
A
WT_LD
LD_KMP-11_KO
LD_KMP-11_KO/KMP-11 complemented
No. of LD Promastigote
DAY1 DAY2 DAY3 DAY4 DAY5 DAY6
B
ns
%of viable parasite
WT_LD
LD_KMP-11_KO
LD_KMP-11_KO/
KMP-11 complemented
C
SSC-A
Metacyclic
35.0
31.5
31.4
LD
KMP-11 KO LD
KMP-11 complemented
KMP-11 KO LD
FSC-A
D
% Metacyclics
WT_LD
KMP-11_KO
KMP11 KO+KMP11 Complemented
E
WT_LD LD>P200
KMP-11
β-actin
25 KD
15 KD
10KD
50 KD
37 KD
F
FA
G
µg CHOL/µg of total protein
MΦ
MΦ+WT_LD
MΦ+LD_KMP-11_KO
MΦ+LD>P200
MΦ+LD_KMP-11_KO/KMP-11 GFP complemented
MΦ+LD_KMP-11_KO +r-KMP-11
MΦ+LD>P200 +r-KMP-11

 © The Author(s)

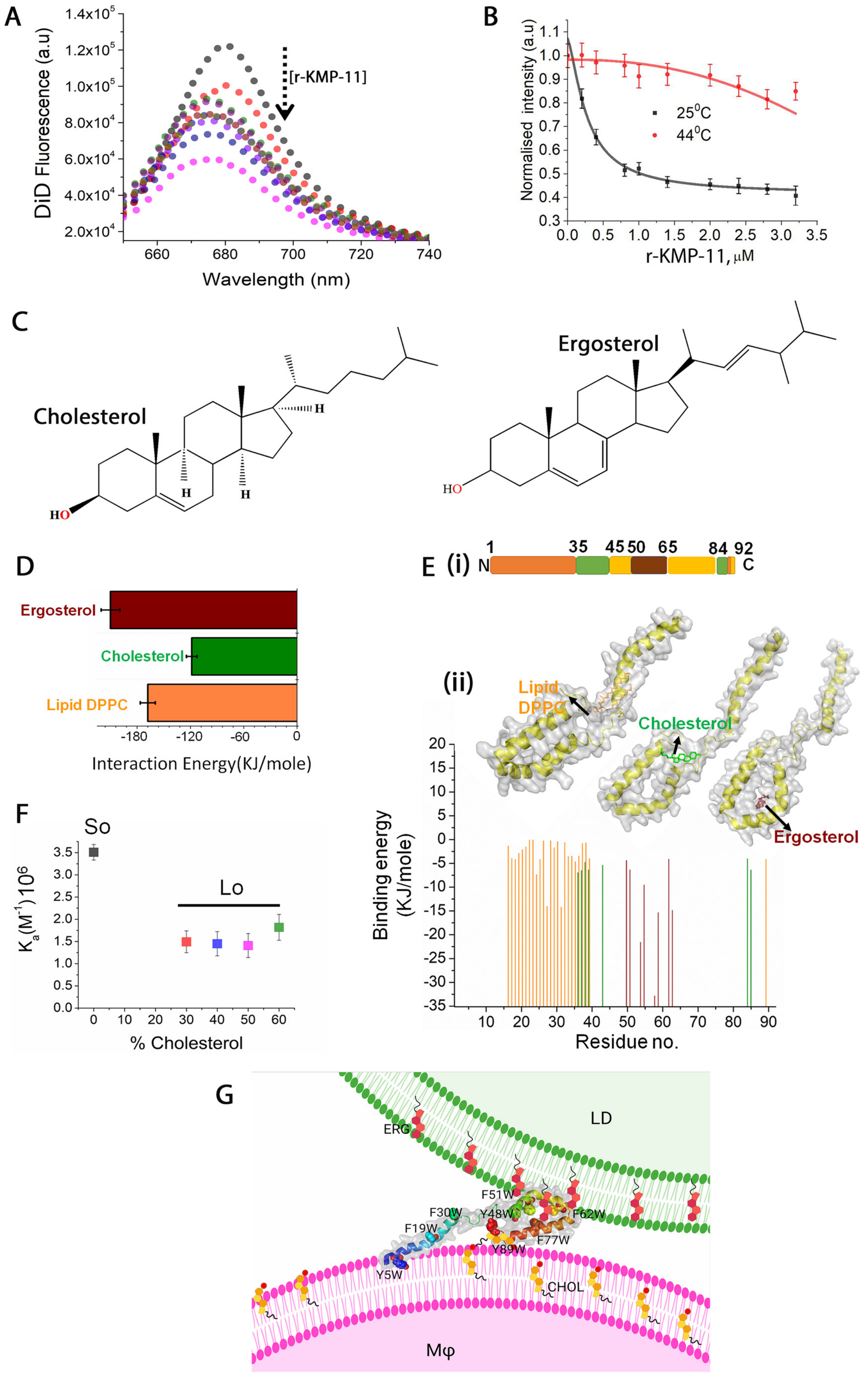
A
DiD Fluorescence (a.u)
[r-KMP-11]
Wavelength (nm)
B
Normalised intensity (a.u)
25°C
44°C
r-KMP-11, μM
C
Cholesterol
Ergosterol
D
Ergosterol
Cholesterol
Lipid DPPC
Interaction Energy(KJ/mole)
E (i)
(ii)
Lipid DPPC
Cholesterol
Ergosterol
Binding energy (KJ/mole)
Residue no.
F
So
Lo
% Cholesterol
G
LD
ERG
F51W
F30W
Y48W
F62W
F19W
F77W
Y89W
Y5W
CHOL
Mφ

Figure EV2. KMP-11 binding with model DPPC SUVs.

(**A**) Representative DiD fluorescence titration spectra shows the binding of r-KMP-11 with DPPC model membranes as was monitored by the decrease in DiD fluorescence intensity (left *y* axis) as well as the blue shifts of the fluorophore (right *y* axis) with increasing concentration of added proteins. (**B**) Plot of normalized membrane embedded DiD fluorophore intensities against increasing WT r-KMP-11 concentration to estimate the binding of r-KMP-11 with DPPC at two different temperatures (25 °C and 44 °C). Two different temperatures were used to see the binding of r-KMP-11 with gel and fluid DPPC membrane. The solid lines indicated the fit of the data to evaluate the binding affinities. Our data showed the binding affinity with Lo/gel phase DPPC (25 °C) is considerably higher than the Ld/fluid phase DPPC membrane (44 °C).The data were expressed as the mean ± SD derived from three independent experiments ($n = 3$) for each temperature group. (**C**) Chemical structures of cholesterol and ergosterol. Region-specific binding of lipid and sterol molecules with KMP-11. (**D**) The binding energy variation of KMP-11 with DPPC lipid, CHOL and ERG as obtained by docking analysis. The data were expressed as the mean ± SD derived from three independent experimental repeats ($n = 3$) for each group. (**E**) A schematic drawing of the overall protein sequence which is marked based on the binding locations of CHOL (green), ERG (brown) and DPPC lipid (orange) as obtained from the molecular docking study. The best posed docked structure of KMP-11 as obtained from docking study was shown. The amino-terminal domain shows the DPPC lipid binding domain, 35–45 AA region and 85–90 AA regions are found to be CHOL binding region, 50–70 AA region is the ERG binding motif. Plot of binding energy of individual lipid and sterol components against the residue number of KMP-11 as obtained from gemdock tool has been shown. Snapshots of the best posed docked structures for CHOL-KMP-11; ERG-KMP-11 and DPPC lipid-KMP-11 as obtained from the molecular docking study. (**F**) Binding of r-KMP-11 with DPPC SUVs containing different mole percentages of CHOL as obtained from the membrane embedded DiD quenching assay. Data indicated a notable decrease in the r-KMP-11 binding with DPPC SUVs containing different percentage CHOL. Here, So and Lo stand for solid order and liquid order states respectively. This data signifies a higher binding affinity of r-KMP-11 towards So domain in comparison to Lo domain. The data were expressed as the mean ± SD derived from three independent experimental repeats ($n = 3$) for each CHOL percentage. (**G**) The plausible orientation of KMP-11 during attachment of the parasite (DPPC-ERG) with MΦ (DPPC-CHOL) membrane.

 © The Author(s)

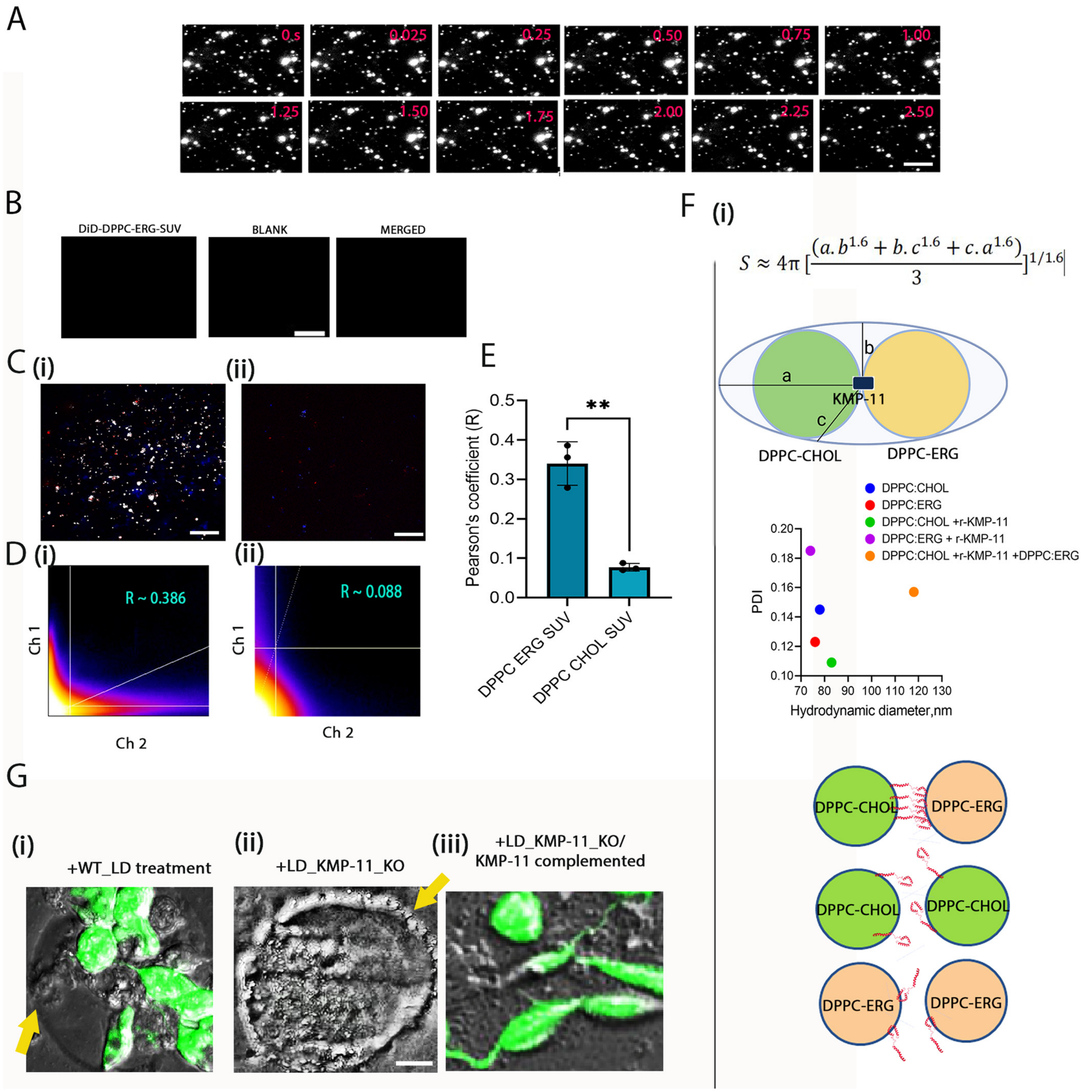
A
0 s
0.025
0.25
0.50
0.75
1.00
1.25
1.50
1.75
2.00
2.25
2.50
B
DiD-DPPC-ERG-SUV
BLANK
MERGED
C (i)
(ii)
D (i)
(ii)
R ~ 0.386
R ~ 0.088
Ch 1
Ch 2
E
Pearson's coefficient (R)
**
DPPC ERG SUV
DPPC CHOL SUV
F (i)
$S \approx 4\pi\left[\frac{(a.b^{1.6} + b.c^{1.6} + c.a^{1.6})}{3}\right]^{1/1.6}$
a
b
c
KMP-11
DPPC-CHOL
DPPC-ERG
DPPC:CHOL
DPPC:ERG
DPPC:CHOL +r-KMP-11
DPPC:ERG + r-KMP-11
DPPC:CHOL +r-KMP-11 +DPPC:ERG
PDI
Hydrodynamic diameter,nm
G
(i) +WT_LD treatment
(ii) +LD_KMP-11_KO
(iii) +LD_KMP-11_KO/ KMP-11 complemented

Figure EV3. The bridging nature of KMP-11.

Co-localization study on PEG cushioned SLB. (**A**) Time lapse images of the DiD-labeled DPPC: ERG SUVs on DPPC: CHOL SLB platform in presence of r-KMP-11. The dimension of each image was kept 32.19×25.94 μm^2. Time lapse images presented here were processed from the continuous recording of Fig. 4Bi. Scale bar is 5 µm. (**B**) TIRF microscopic images of DiD-labeled DPPC ERG SUVs (red channel), blank (blue channel) and the merged image. Scale bar is 10 µm. (**C**) (i) Colocalised micrograph of DiD-labeled DPPC ERG SUVs (red channel) and Alexa-488 maleimide labeled r-KMP-11 (blue channel). Scale bar is 10 µm. (ii) colocalised micrograph of DiD-labeled DPPC CHOL SUVs (red channel) and Alexa-488 maleimide labeled r-KMP-11 (blue channel). Scale bar is 10 µm. (**D**) Scatter plots of colocalisation of (i) DiD-labeled DPPC ERG SUVs (red channel) and Alexa-488 maleimide labeled r-KMP-11 (blue channel) system and (ii) DiD-labeled DPPC CHOL SUVs (red channel) and Alexa-488 maleimide labeled r-KMP-11 (blue channel). The R (pearson's coefficient) values stand for the degree of colocalisation. (**E**) Plot shows the values of pearson's coefficients obtained from TIRF imaging using DPPC:CHOL and DPPC: ERG SUVs. Data were presented as mean ± SD derived from three independent experiments for both groups. The level of significance has been estimated using unpaired *t* test in GraphPad Prism (version 9) application. **P value = 0.0012. (**F**) (i) Dynamic light scattering (DLS) study to estimate the hydrodynamic radii of different sterol-containing SUVs. We observed comparable hydrodynamic radii for DPPC-ERG and DPPC-CHOL. Hydrodynamic radius increased significantly when we added r-KMP-11 to the equimolar mixture of DPPC-ERG and DPPC-CHOL. Implying ellipsoid approximation while two vesicles are attached by a KMP-11 bridge, we calculated the average surface area of the combined system, which was found to be ~43,870.42 nm^2. Interestingly, we found that our calculated surface area matched well with the surface area obtained from DLS data for DPPC-ERG + DPPC-CHOL+r-KMP-11 system (observed surface area ~41,094 nm^2), which further suggests that KMP-11 can act as a bridging molecule between CHOL and ERG rich membranes. Here, PDI stands for poly-dispersity index. (ii) Different orientations of KMP-11 in SUV environment in presence of CHOL and ERG in SUVs. (**G**) Images representing interaction of CFSE labeled WT_LD and LD_KMP-11_KO and complemented LD lines on supported Lipid Bilayer (SLB) composed of DPPC CHOL 30%. Significant attachment of (i) WT_LD parasites was observed on lipid surface which is absent for (ii) LD_KMP-11_KO parasites. Attachment is restored for (iii) LD_KMP-11_KO/complemented parasites on SLB surface. The yellow arrows indicate the edge of the supported lipid bilayer. Scale bar is 1 µm. Source data are available online for this figure.

 © The Author(s)

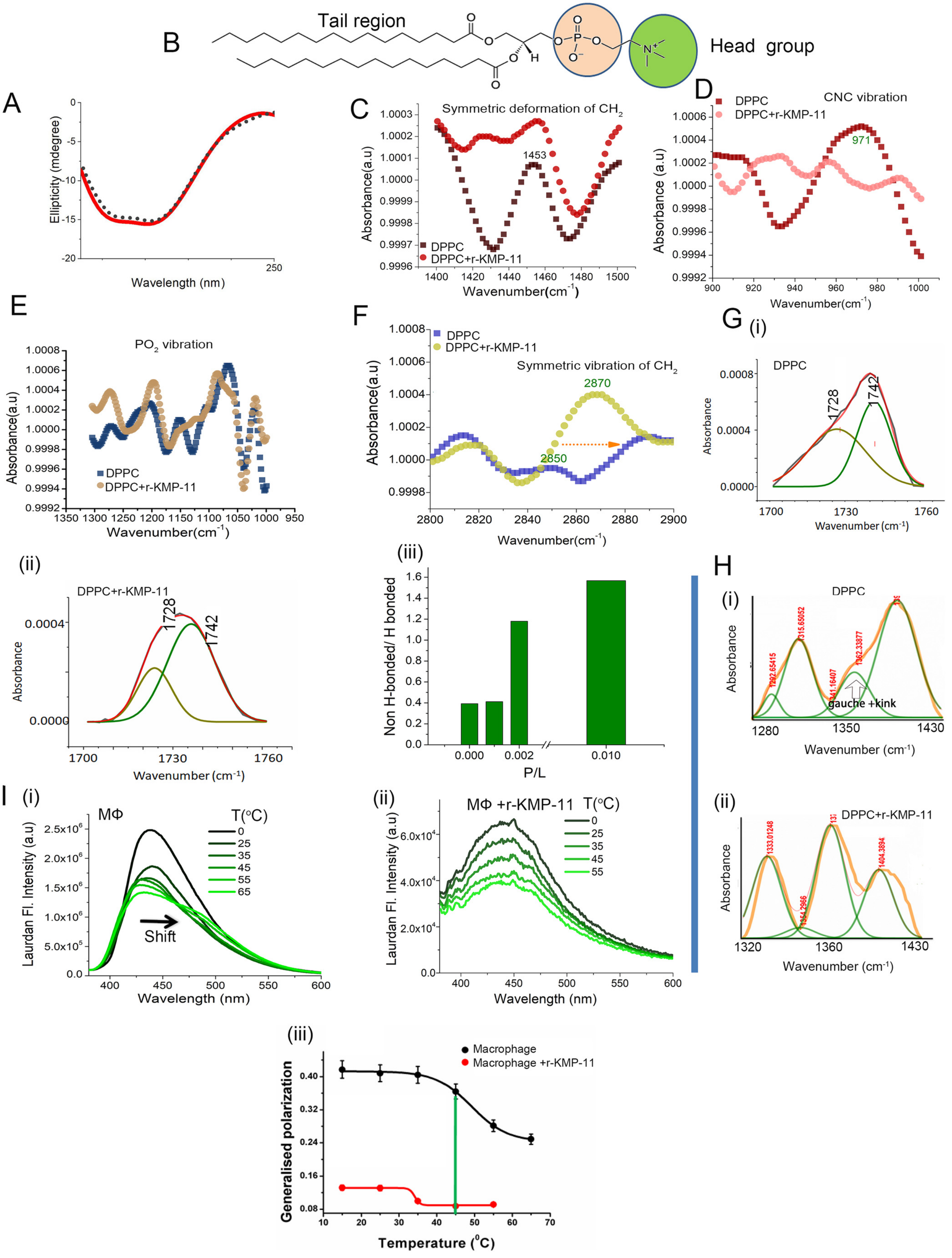
A
B
Tail region
Head group
C
Symmetric deformation of CH_2
DPPC
DPPC+r-KMP-11
D
CNC vibration
E
PO_2 vibration
F
Symmetric vibration of CH_2
G (i)
DPPC
(ii)
DPPC+r-KMP-11
(iii)
Non H-bonded/ H bonded
P/L
H
(i)
DPPC
gauche +kink
(ii)
DPPC+r-KMP-11
I (i)
MΦ
T(°C)
Shift
(ii)
MΦ +r-KMP-11 T(°C)
(iii)
Macrophage
Macrophage +r-KMP-11
Generalised polarization
Temperature (°C)

Figure EV4. Interactions induced protein–lipid conformational perturbations.

(**A**) Far UV-CD spectra of r-KMP-11 in the absence and presence of DPPC membrane. No significant conformational change in the secondary structure of r-KMP-11 due to membrane binding was evident from the CD data. Membrane perturbations study due to KMP-11-lipid binding. (**B**) The molecular structure of DPPC lipid in which the head group and tail group regions are marked. FTIR signatures of (**C**) C-N-C, (**D**) PO_2. (**E**) Symmetric deformation of CH_2. (**F**) Symmetric vibrations of CH_2, both in absence and presence of r-KMP-11. (**G**) Deconvolution of carbonyl (C = O) stretching vibrations in (i) absence and (ii) presence of r-KMP-11 to measure the population of hydrogen bonded carbonyl frequency (appears at 1728 cm^{-1}) and nonhydrogen bonded frequency (at 1742 cm^{-1}). Interestingly, r-KMP-11 binding increased the population of nonhydrogen bonded carbonyl frequency in a concentration-dependent manner as shown in (iii). Moreover, enhanced nonhydrogen bonded vibrational states due to r-KMP-11 binding also indicated bilayer thinning. (**H**) Deconvoluted FTIR spectral signatures of the CH_2 wagging band frequency of DPPC in absence (i) and the presence (ii) of r-KMP-11. This FTIR data suggested the increase of gauche rotamers of DPPC due to protein binding. (**I**) Measurement of MΦ membrane fluidity in the absence and presence of KMP-11. Plot of laurdan emission intensity of labeled MΦ with increasing temperature in the (i) absence and (ii) presence of r-KMP-11. (iii). Generalized polarization (GP) of laurdan labeled macrophage membrane with respect to temperature in absence (black) and presence (red) of r-KMP-11. Typically, concentration of r-KMP-11 was taken 50 μM. The data were represented as the mean ± SD derived from three independent GP experiments ($n = 3$) for each temperature.

© The Author(s)
